# Dysbiotic shift in the oral microbiota of patients with Alzheimer's disease compared to their healthy life partners—a combinatorial approach and a paired study design

**DOI:** 10.1186/s13195-025-01941-1

**Published:** 2026-01-13

**Authors:** Christian Weber, Daniel Wind, Patrick Petzsch, Tillmann Supprian, Alexander Dilthey, Julia Christl, Patrick Finzer

**Affiliations:** 1 Institute for Medical Microbiology and Hospital Hygiene, University Hospital Düsseldorf, Heinrich Heine University Düsseldorf, Universitätsstrasse 1, Düsseldorf, 40225 Germany; 2 Department of Psychiatry, Medical Faculty, LVR-Clinic Düsseldorf, Heinrich-Heine University Düsseldorf, Bergische Landstraße 2, Düsseldorf, 40629 Germany; 3 Genomics and Transcriptomics Laboratory (GTL), Biological and Medical Research Center (BMFZ), Medical Faculty and University, Hospital Düsseldorf, Heinrich Heine University Düsseldorf, Universitätsstrasse 1, Düsseldorf, 40225 Germany; 4 Department of Psychiatry and Psychotherapy, Medical Faculty, LVR-Clinic Essen, University of Duisburg-Essen, Virchowstraße 174, Essen, 45147 Germany

**Keywords:** Alzheimer, Dementia, Spouses, Oral microbiota, Next generation sequencing, Periodontal disease, Metagenomic

## Abstract

**Background:**

The oral microbiota has been associated with Alzheimer's disease (AD). However, earlier studies provided conflicting results using varying sampling methods, sequencing techniques, and statistics, as well as independent subjects.

**Methods:**

To robustly identify disease-associated microbial features, we recruited patients and their healthy life partners from the same households sharing a more similar microbiota compared to independent individuals increasing statistical power via paired design and combined three different sequencing methods – including metagenomics—and several bioinformatic pipelines. We recruited 26 AD-patients and their life partners. Salivary and supragingival samples were collected and a clinical examination of the mouth was performed.

**Results:**

Both groups showed comparable oral health. By focusing primarily on recurrently identified species across the different datasets we were able to identify a Core dysbiosis. This Core dysbiosis surprisingly spares the most central of oral diseases pathogens, namely *Porphyromonas gingivalis*. However, it includes numerous other species commonly associated with oral pathologies such as *Prevotella nigrescens, Streptococcus anginosus, Dialister invisus, Anaeroglobus geminatus, Olsenella uli* and *Mogibacterium timidum*. In contrast, more host-compatible species such as *Prevotella melaninogenica* or *Streptococcus parasanguinis* are identified in controls.

**Conclusions:**

This is the first study using a combined sequencing approach and a paired study design to identify robust features of the oral microbiota of AD-patients. Although promising, the results should nevertheless be interpreted with caution, as the cross-sectional study design limits the possibilities of interpretation, and larger, longitudinal data are necessary for causal conclusions. However, this combined approach on multiple processing levels to identify intra-partnership differences still offers the possibility to better identify disease-associated microbial features potentially involved in AD-pathogenesis.

**Trial registration:**

This study was prospectively registered at the German Clinical Trials Register (DRKS00023456) at the 30th of November 2020.

**Supplementary Information:**

The online version contains supplementary material available at 10.1186/s13195-025-01941-1.

## Introduction

Alzheimer's disease (AD) is the most common form of dementia. Due to increasing life expectancy, the number of individuals with dementia could reach 150 million globally by the year 2050 [1]. AD is characterized by a progressive decline in cognition and the presence of amyloid β−40/−42 and tau proteins accompanied by neuroinflammation, activation of microglia and mitochondrial dysfunction [2].

AD is increasingly recognized as a multifactorial disease, influenced by genetic, cardiometabolic and environmental factors [3, 4]. Alongside other factors, increasing evidence for the connection between the oral cavity and AD is emerging. Patients with periodontal disease (PD) were found to possess an increased risk of developing AD [5], with an association between PD and brain amyloid-β load [6] and cognitive decline [7]. Oral dysbiosis, defined as 'perturbation to the structure of complex commensal communities' leading to diseases [8], is thereby assumed to contribute to PD [2, 9].

Microbiota of the oral cavity are therefore emerging as possible drivers for the development of AD [10]. For this reason, changes in oral microbiota composition, microbial metabolites and bacterial components have been investigated in patients with AD, but with widely varying methods and inconsistent results: Although all studies investigating the oral microbiota in AD identified differences between patients and controls it remains difficult to identify clear trends [2, 11–13].

Multiple lines of evidence connect the oral microbiome with the pathophysiology of AD. This includes direct as well as indirect connections: For example, it is well described that periodontal inflammation is associated with a higher systemic inflammatory burden [14]. This systemic inflammation increases neuroinflammation [15] and contributes to disruption of the blood brain barrier [16], both common features more and more recognized as part of the AD pathophysiology [2]. Alternatively, or in parallel, PD increases the risk of cardiometabolic diseases [17] which are known risk factors for AD [3, 4].

Another, much more direct connection of the oral microbiome and AD is the long known periodontal bacterium *Porphyromonas gingivalis* (PG), a member of the so-called Red Complex, describing the most central PD pathogens [18, 19]. PG has been detected in the cerebrospinal fluid and saliva of AD-patients [20]. Post-mortem studies identified PG-DNA, lipopolysaccharides (LPS), and its protease gingipain in AD brains, correlating with tau pathology [20, 21]. Animal experiments show that oral or systemic PG exposure induces cognitive deficits, neurodegeneration, and Aβ accumulation [22, 23]. Treatment with the gingipain inhibitor COR388 reduced Aβ deposition and neuroinflammation in mice [20], though human trials were halted due to hepatotoxicity [24, 25].

PG has been described as a keystone-pathogen of the oral cavity, able to disrupt oral microbial homeostasis and drive PD even in low abundances [26]. PG inhibits innate immune mechanisms, fostering overgrowth of other microbes and initiating a dysbiotic shift [27]. This imbalance triggers chronic inflammation, proteolytic tissue destruction, and nutrient release [28]. In mice, PG induces dysbiosis and bone loss even at low concentrations (< 0.01%) yet fails to cause disease in germ-free or complement receptor–deficient animals, underscoring the importance of microbial synergy and innate immunity [29].

Potential mechanisms linking PG to AD include systemic inflammation and neuroinflammation [14, 15], yet direct CNS invasion remains unproven, as only bacterial components, not living PG, have been detected [30]. An emerging model centers on PG’s outer membrane vesicles, which carry gingipains, LPS, and genetic material and may cross the blood–brain barrier due to their nanoscale size [31, 32]. Experiments confirm that oral administration of PG-derived vesicles can induce neuroinflammation, tau phosphorylation, and memory impairment, with fluorescence imaging showing their presence within the brain [33]. This collective evidence identifies PG as a potential key driver of oral dysbiosis and related diseases.

One challenge when investigating these potential connections especially in a clinical context is the bidirectional connection of oral health and dementia: It is well documented that patients with cognitive deficits commonly present with poor oral health due to increasing deficits in daily tasks [34]. Therefore, it is difficult to identify a potential disease contribution of oral health and/or the oral microbiome to the initiation or progression of AD making a coherent study design even more important.

The oral cavity presents approximately 700 species of microorganisms [35]. Several distinct habitats exist in the oral cavity, including, but not limited to, buccal mucosa, sub- and supragingival plaques and saliva [36]. To capture these niches while getting an impression of the oral microbiota as a whole, we combined salivary and supragingival samples, since saliva is relatively independent of a specific anatomical niche while supragingival samples potentially reflect a localized process [37].

Microbiome studies are known to be susceptible to methodological limitations. These include, among other factors, differences in sequencing platforms and target regions. Broadly speaking, next-generation sequencing (NGS) approaches can be divided into short- and long-read sequencing, sometimes also referred to as second- and third-generation sequencing, as short-read technologies represent the older generation. As the names suggest, the primary difference between these technologies lies in the length of the sequencing reads: Short-read platforms typically produce reads of approximately 150–800 bp, whereas long-read technologies generate reads 10,000 bp and longer. Theoretically, these longer reads are better suited to capture repetitive or complex genomic regions, although they were, at least in their early stages of development, more prone to sequencing errors. Consequently, short-read sequencing is often considered more robust and cost-effective but provides a more limited level of information. Long-read-sequencing on the other hand potentially offers more information but is still in its infancy and not as widespread available as short-read-technologies [38].

Beyond technological considerations, the two principal sequencing approaches in this field are 16S rRNA gene sequencing and metagenomic, or whole-genome, sequencing. The 16S rRNA gene encodes the small ribosomal subunit and serves as a ubiquitous bacterial marker gene. It is typically present in all bacteria and contains nine variable regions, commonly referred to as V1–V9. Sequencing specific variable regions of this approximately 1,500 bp-long gene enables the identification of bacterial taxa based on recognition and amplification of a comparatively small target sequence, thereby massively increasing the effective DNA 'yield' [39].

However, despite its efficiency, the 16S approach has several inherent limitations. Because only parts of the 16S gene are sequenced, little to no functional information can be derived from the data. Moreover, while the 16S gene is an effective target for detecting bacterial DNA, it cannot capture fungi or viruses, which also constitute important components of the microbiome [40]. Additional challenges include bacterial species with multiple 16S gene copies, effectively influencing abundance estimation [39]. Also, 16S-based approaches typically require amplification steps, which can artificially bias the relative abundance of highly prevalent taxa [41, 42].

Metagenomic sequencing therefore serves as an alternative approach to 16S rRNA gene sequencing. Metagenomics refers to the sequencing of all genetic material present in a sample. In the context of microbiome research, this includes not only bacterial DNA but also fungal, viral, and particularly host DNA. As metagenomic sequencing typically omits an amplification step, it is considered more capable of capturing the true composition of the sampled microbiome. However, achieving sufficient sequencing coverage typically requires larger amounts of input material. This can be especially challenging for mucosal samples, which are often dominated by host DNA, resulting in comparatively lower sequencing depth for bacterial reads than amplification-based approaches [43, 44].

Thus, sequencing the microbiome generally involves a trade-off between methodological sensitivity, sequencing depth, and associated costs. While the identification of bacteria based on sequencing of the 16S rRNA gene is an established method, it is nevertheless prone to among other things amplification bias leading to overrepresentation of highly abundant taxa, probably not ideally reflecting the real composition of the ecosystem of interest [38, 41, 42]. Metagenomic sequencing on the other hand should overcome these shortcomings but has the problem of potentially extremely small numbers of bacterial reads due to mainly containing host-DNA [43, 44].

It has also been observed that animals and people living in the same households share a more similar microbiota compared to independent people [45, 46]. This effect goes so far that even siblings with shared genetic backgrounds tend to show less similarities in their microbiota than spouses [47]. This so-called co-housing effect together with a higher probability of life partners sharing cardiometabolic risk factors and health behaviors [48, 49] increases the chance that microbial differences are truly disease-associated. Especially in case of smaller sample sizes such natural pairing can be useful to increase statistical power, with some authors even recommending to better opt for smaller* n* of paired samples compared to larger *n* of independent samples in case of differential expression studies [50]. For this reason, we recruited AD-patients together with their healthy life partners as controls to focus on these potentially more robust intra-partnership differences.

Therefore, this work tries to achieve a more robust understanding of the oral microbial differences between AD-patients and controls and overcomes some of the drawbacks of earlier work. Some aspects distinguishing this work therefore include: 1) The examination of AD-patients and their life partners as controls with similar microbiome, environmental conditions and levels of oral health, 2) the investigation of patients at an early disease stage, increasing the chance of capturing a disease-associated microbial pattern before the patients capability for oral care decreases, 3) the biochemical confirmation of the AD diagnosis in contrast to clinical diagnosis of dementia only, 4) the combination of three different sequencing methods, including, to the best of our knowledge, one of the first metagenomic approaches in the context of oral microbiota and neurodegenerative diseases, 5) the combination of several bioinformatic pipelines for taxonomical classification and differential abundance analysis and 6) the focus on recurrently identified taxa for a more robust differential abundance analysis.

## Materials and methods

**Fig. 1:**
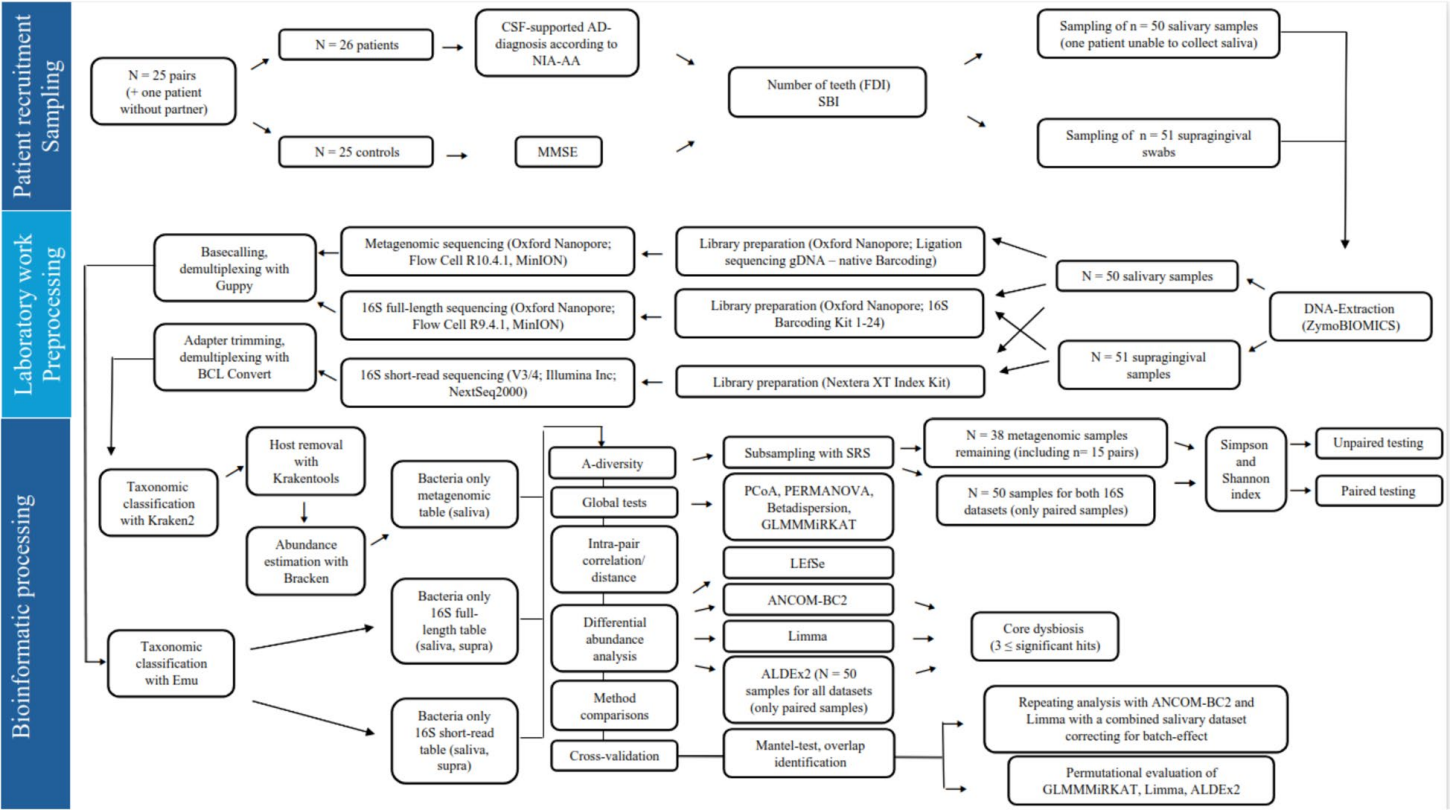
Flowchart illustrating the overall workflow of the study divided by patient recruitment and sampling, laboratory work and preprocessing and the final bioinformatic processing of the data. If not shown otherwise all samples were included in the according analysis step. Abbreviations: AD, Alzheimer's disease; ALDEx2, ANOVA-Like Differential Expression 2; ANCOM-BC2, Analysis of Compositions of Microbiomes with Bias Correction 2; Bracken, Bayesian Reestimation of Abundance with KrakEN; CSF, Cerebro-spinal-fluid; DNA, Deoxyribonucleic acid; FDI, Fédération Dentaire Internationale; gDNA, Genomic DNA; GLMMMiRKAT, Generalized Linear Mixed Model Microbiome Regression-based Kernel Association Test; LEfSe, Linear Discriminant Analysis Effect Size; Limma, Linear Models for Microarray; MMSE, Mini Mental Status Examination; NIA-AA, National Institute of Aging and Alzheimer’s Association; PCoA, Principal Coordinate Analysis; PERMANOVA, Permutational Multivariate Analysis Of Variance; SBI, Sulcus bleeding index; SRS, Scaling with Ranked Subsampling; Supra, Supragingival; V3/V4, Variable region 3/4

An overview of all following clinical, laboratory, and bioinformatical processing steps, including the exclusion of samples from individual analyses, is given in Fig. 1.

### Setting and subjects

Patients with mild AD were recruited by the Department of Psychiatry of the LVR-Klinikum Düsseldorf. Cerebro-spinal-fluid biomarker supported diagnosis of probable AD was established according to the revised National Institute of Aging and Alzheimer’s Association (NIA-AA) criteria [51, 52]. Spouses or partners, who lived in the same household, were recruited as healthy controls. However, one patient (patient02) came without a partner while another patient (patient26) was unable to collect enough saliva. These samples were included as far as possible but had to be left out from several analysis steps to guarantee the paired design. Cognitive status was evaluated with the Mini Mental Status Examination (MMSE) [53], with a minimum sum score of 20 points for the patients and 27 points for the controls. Exclusion criteria were acute infections of the upper respiratory tract or oral cavity (e.g., stomatitis), severe impairment of general condition as well as patient history with substance use disorder, stroke, multiple sclerosis, epilepsy, Parkinson's disease, or schizophrenia. All subjects gave written informed consent for participation. Patients had a preserved ability to give informed consent. The study was approved by the ethics committee of the medical faculty of the Heinrich-Heine-University (no.: 2020-1155_1) and was performed in accordance with the ethical standards as laid down in the 1964 Declaration of Helsinki and its later amendments.

### Examination of oral status

The oral status was obtained by a physician which had been trained by a dentist for this purpose. All examinations were performed by the same person and at the same location. As an easy, yet objective and reproducible way to investigate oral health and oral health behavior we counted the patients' number of teeth as an indicator of earlier periodontal disease [54], performed the modified sulcus bleeding index (SBI) as a measurement of current periodontal disease and used a simple questionnaire regarding the patients' dental history.

The number of teeth was determined according to the internationally recognized scheme of the Fédération Dentaire Internationale (FDI) [55]. The modified sulcus bleeding index (SBI) according to Lange et al. [56] was used to objectively examine the gingival tissue for the presence of bleeding as an expression of local inflammation.

### Sampling

The sampling of all participants was performed by the same investigator. At the time of the sampling, the participants should not have eaten, drunk anything but water, brushed their teeth, used dental floss or mouthwash, chewed gum or consumed throat lozenges for two hours beforehand. Compliance with these measures was asked before the samples were taken. One supragingival swab and a saliva sample were taken; for the saliva samples, the subjects were asked to collect saliva in their mouths and then dispense it using the mouthpiece provided on the saliva collection aid. All probe vessels contained DNA/RNA Shield solution which is designed to allow transport and storage at room temperature (Zymo Research Europe, Freiburg, Germany).

Salivary samples were used for metagenomic, 16S full-length and 16S short-read sequencing, while supragingival swabs were only sequenced with both 16S approaches. No repeated measurements were taken.

### DNA extraction, library preparation and sequencing

The ZymoBIOMICS DNA/RNA Miniprep Kit (Zymo Research Europe, Freiburg, Germany) was used to extract the DNA for all subsequent sequencing steps according to the manufacturer's instructions. The DNA concentration was determined using a Qubit 3.0 Fluorometer (Thermo Fisher Scientific, Germany) and the Qubit 1X dsDNA High Sensitivity Assay Kit (Thermo Fisher Scientific, Germany).

Samples were sequenced using three different techniques: Short-read based targeted sequencing of the 16S variable regions V3 and V4, long-read-based full-length 16S sequencing, and long-read-based metagenomic sequencing.

Amplification and library preparation of the variable regions V3 and V4 of the 16S rRNA gene was performed according to the Illumina 16S metagenomics protocol (Part #15,044,223 Rev. B) using the Nextera XT Index kit (Illumina Inc. San Diego, CA, USA). Bead purified libraries were normalized, pooled, and finally sequenced on the NextSeq2000 system (Illumina Inc. San Diego, CA, USA) with a read setup of 2 × 300 bp. The BCL Convert Tool (version 4.0.3) was used to convert the bcl-files to fastq-files as well as for adapter trimming and demultiplexing.

For library preparation for full-length 16S sequencing, the 16S Barcoding Kit 1–24 (Oxford Nanopore Technologies, United Kingdom) was used according to the manufacturer's instructions with 10 ng of DNA as starting point. In this protocol, the complete 16S rRNA gene from previously extracted DNA is first amplified by PCR. The primers contain barcodes for sample assignment, as well as 5'tags, which later allow sequencing adapters to be bound without ligase. Again, after purification the samples are pooled and sequencing adapters for binding to the eponymous pore in the flow cell are linked. A Flow Cell (R9.4.1) (Oxford Nanopore Technologies, United Kingdom) in a MinION sequencing device (Oxford Nanopore Technologies, United Kingdom) was used for sequencing according to the manufacturer's instructions with a minimal length of 200 bp and high accuracy base calling.

For library preparation for metagenomic sequencing, the Ligation Sequencing gDNA – native barcoding Kit (Oxford Nanopore Technologies, United Kingdom) was used according to the manufacturer's instructions with 400 ng of DNA as starting point. In this protocol, previously extracted genomic DNA is first end repaired and then an adenine is attached to the 3' end (dA tailing) allowing the ligation of individual barcode sequences for later sample assignment. After subsequent purification, the samples are pooled to finally ligate sequencing adapters that enable the DNA-fragment to bind to the eponymous pore. A Flow Cell (R10.4.1) (Oxford Nanopore Technologies, United Kingdom) in a MinION sequencing device was used for sequencing according to the manufacturer's instructions with a minimal length of 20 bp and high accuracy base calling.

For both Nanopore sequencing approaches data was acquired with MinKNOW Core (version 5.4.7). Basecalling and demultiplexing was done using Guppy (version 6.4.6). Demultiplexed fastq.gz-files of the same barcode were combined to one fastq-file.

### Bioinformatic and statistical analysis

Starting with the raw sequencing results as fastq-files, the data were processed in the package and environment management system Conda (version 4.13.0). Most of the statistical operations were performed in R (version 4.3.1). Custom python-scripts were used in Python (version 3.7.3). Unless otherwise stated, default settings were used, and recommended standard databases were built. Software packages used with Conda, R, or Python will be referred to as packages, libraries, and modules according to the appropriate terminology. Necessary metadata files for the analyses are included in the supplementary material.

#### Taxonomical classification

For taxonomical classification, the package Emu (version 3.4.5) was used for the short-read 16S sequencing and full-length 16S sequencing data [57]. As a reference, Emu uses a combined database of the two databases rrnDB v5.6 [58] and NCBI 16S RefSeq [59] with 49,301 sequences from 17,555 bacterial and archaea species. Relative abundances and absolute read counts were calculated using the basic command 'emu abundance –keep-counts sequences.fastq' with the option '–type sr' for the short-read 16S data. With the command 'emu combine-outputs table_rank.txt rank' with and without the option '–counts' tables containing the relative abundances and absolute read counts of all samples for the different phylogenetic ranks from species to phylum were created [57]. The tables created this way were used for further statistical analysis.

The metagenomic sequencing data were classified using the package Kraken2 (version 2.1.1) with the basic command 'kraken2 –db database_name –report kraken_report_file.txt –output kraken_read_file.txt sequences.fastq' [60]. For this purpose, Kraken2 also uses NCBI RefSeq as a database [59]. After an initial classification of all reads with Kraken2 we used the script extract_kraken_reads.py provided with the package krakentools (version 1.2) via the command 'python extract_kraken_reads.py -k kraken_read_file.txt -r kraken_report_file.txt -s sequences.fastq -t 2 –include-children -o sequences_bacteria_only.fastq ' to extract reads belonging to the superkingdom 'Bacteria' and save these in a new fastq-file [61]. These were then used for a second analysis with Kraken2.

The exact determination of the abundances was then carried out using the package Bracken (version 2.6.0) with the kraken_report_file.txt produced by Kraken2 as input using the basic command 'bracken -d database_name –i kraken_report_file.txt -r 500 –l rank –t 3' to produce files with relative abundances and absolute read counts for the different phylogenetic ranks from species to phylum. A read length of 500 and a threshold of three reads for identification of a taxon were set as parameters. To create tables with relative abundances and absolute read counts of all samples the script combine_bracken_output.py contained in Bracken was executed with the output files of the prior step as input via the command 'combine_bracken_outputs.py –files *rank.bracken -o table_rank.txt' [62]. The tables created this way were used for further statistical analysis.

#### Intra-pair correlation and Bray–Curtis-distance

We first wanted to test our initial assumptions of pairs having a more similar oral microbiota than controls. For this, we used the relative bacterial abundances of partners to calculate Pearson- and Spearman-correlation and Bray–Curtis-distances in R using the vegan (version 2.6.8) and tidyverse (version 2.0.0) libraries. We then used a permutation test to iteratively calculate correlation and distance between random samples and compare the mean correlation and distance to the true intra-pair results. For the species- and genus-level we also repeated this with filtered data using only the top 100 most abundant taxa (see Supplementary scripts).

#### Α-diversity

To quantify α-diversity, absolute read counts for bacterial species were subsampled in R using the Scaling with Ranked Subsampling (SRS) library (version 0.2.3). For the full-length and short-read 16S data, the sample with the lowest sequencing depth was identified with the SRS shiny app and then selected for 'Cmin'. For the metagenomic data, which showed significantly lower read counts after filtering eukaryotic and viral DNA, a minimum of 1,000 reads was set for normalization as this number is mentioned as the lowest reliable read count by the authors [63]. This meant that 12 of the 50 samples were not included in the determination of α-diversity (seven patients, five controls). Full-length and short-read salivary data were subsampled to their lowest read count of 22,945, respectively 768,222 reads, while full-length and short-read supragingival data were subsampled to 2,626 and 640,906 reads. Using these subsampled data, the Simpson and Shannon indices were determined in R using the vegan library.

All α-diversity indices were then tested for normality and homogeneity of variance via Shapiro-Wilks-test and Levene-test and accordingly tested for between group differences with parametric or nonparametric tests using the car (version 3.1.3) and dplyr (version 1.1.4) libraries. For short-read and full-length data we only used paired samples leaving out patient02 and control26 in case of salivary samples, respectively only patient02 for supragingival samples to then use T-test for dependent samples respectively Wilcoxon-signed-rank-test. As 12 samples were left out from the metagenomic data, we decided to test them in two ways: First we used the 38 subsampled samples and tested them in disregard of their pairing with T-test for independent samples respectively classical Wilcoxon-test. Then we identified all remaining complete pairs in the subsampled samples, effectively further reducing the number of samples to 30 consisting of 15 pairs, to then use T-test for dependent samples respectively Wilcoxon-signed-rank-test (see Supplementary scripts).

#### Β-diversity

For β-diversity, the Bray–Curtis-dissimilarity and Principal Coordinate Analysis (PCoA) were determined in Python using the modules sklearn.manifold (1.2.2), scipy.spatial (1.10.0) and matplotlib (version 3.7.1). In addition, a Permutational Multivariate Analysis Of Variance (PERMANOVA) and a Betadispersion-test were performed based on Bray–Curtis-dissimilarity in R using the vegan library to test for a difference in the centroids and dispersion of the groups. Block-wise permutations were used to account for the paired design while age, sex, BMI (Body-Mass-Index), smoking status, number of teeth and SBI were incorporated as covariates (see Supplementary scripts).

We also used the Generalized Linear Mixed Model Microbiome Regression-based Kernel Association Test (GLMMMiRKAT) function of the MiRKAT library (version 1.2.3) in R based on Bray–Curtis-, Jaccard- and Manhattan-distances calculated with the vegan library. MiRKAT generally uses a regression model based on kernel-matrices generated from varying distance metrics to predict associations between microbiome data and among others a binary outcome. GLMMMiRKAT additionally uses a generalized mixed model allowing for dependence among samples. In this case, the participant state as patient or control served as a binary outcome for the analysis and the partner pairing as a clustering vector. P values for the individual distances as well as the combined so-called omnibus value were calculated via permutation. Again, age, sex, BMI, smoking status, number of teeth and SBI were incorporated as covariates. We also used GLMMMiRKAT to identify associations between the microbial data and the number of teeth, SBI, smoking status, and BMI switching these variables with the group as the primary outcome [64] (see Supplementary scripts).

#### Differential abundance analysis

All in all, we used four different methods for differential abundance analysis: Linear Discriminant Analysis Effect Size (LEfSe), Analysis of Compositions of Microbiomes with Bias Correction 2 (ANCOM-BC2), Limma (Linear Models for Microarray) and ALDEx2 (ANOVA-Like Differential Expression 2). These methods were used individually to identify differentially expressed taxa to then identify the overlap between ANCOM-BC2, Limma and ALDEx2 as the most robust differences across all three result sets. LEfSe on the other hand was used independently and its results were only included as a form of cross-validation of the other results (see Discussion and Table 2).

With ANCOM-BC2, Limma and ALDEx2 we used Benjamini–Hochberg as the multiple testing correction method with a less strict cut-off for adjusted P of 0.1. We hypothesize that by focusing only on the frequently identified taxa in the next step, a useful compromise between control of false-discovery-rate with small sample size and avoidance of overadjustment could be achieved. All analyses were performed for all taxonomical levels from species to phylum.

The relative abundances were first analyzed with the package LEfSe (version 1.1.2) and the results were processed graphically. LEfSe identifies and weighs traits that are most likely to determine the difference between two or more biological states. The process is based on three steps: A Kruskall-Wallis-rank-sum-test to identify significantly different taxa, followed by pairwise Wilcoxon-rank-sum-tests of possible subgroups, if any. The significance level was set by us to the recommended default of 0.05. In the third step, the effect size of the previously identified characteristics is determined using linear discriminant analysis. The logarithm to the base 10 of the result then gives the so-called LDA score (Linear Discriminant Analysis). An LDA score of 2.0 was selected as the threshold value for the effect size of a relevant characteristic in accordance with the standard recommendations. The characteristics are then presented in order of effect size. To perform the analysis, the scripts format_input.py, run_LEfSe.py and LEfSe_plot_res.py contained in the package were executed using a.txt-file as input and setting the parameters for the initial script to '-c 1 –s –1 –u –1 –o 1,000,000' [65].

As the second method, absolute read counts were analyzed in R using ANCOM-BC2 with the ANCOM-BC library (version 2.4.0). ANCOM-BC2 initially discards taxa that fall below a defined prevalence in the samples and does not include them in the actual analysis. The standard value of 0.1 was selected as the limit value for the prevalence. A logarithmic transformation of the read numbers is then performed, whereby zero values are considered missing. In the next step, sample-specific and taxon-specific bias are estimated and corrected. Sample-specific biases are essentially different sequencing depths of the samples. The correction of taxon-specific bias works with the basic assumption that most taxa are not differentially abundant. The log-transformed data is then used to create a linear model, where the bias are subtracted from the respective coefficients and bias-corrected estimates are obtained. The actual hypothesis tests, including FDR control, can then be performed and the result presented as a log-fold-change with P value and adjusted P value. This is followed by an assessment of the robustness of the results against different pseudo-counts, which are used in place of the zero values. No pseudo-counts are used for the actual analysis. However, by testing pseudo-counts from 0.01 to 0.5 in ascending order to determine whether they influence the identification of the corresponding taxon as statistically significant, an additional criterion for the robustness of the results is obtained if a taxon remains statistically significant across all pseudo-counts [66, 67].

A special feature of the analysis using ANCOM-BC2 is the separate identification of structural zeros. In the simplest case, a structural zero is a taxon that does not occur at all in one group but does occur in the other. Corresponding taxa are treated as separate results and are not included in the actual analysis, which further reduces the number of taxa to be analyzed. While these taxa could be defined as being statistically different, we did not include them in our Core analysis as most of them were only identified in a few subjects.

To perform the analysis with ANCOM-BC2 a phyloseq object is created with the Phyloseq library (version 1.46.0) containing absolute read counts and metadata identifying the samples as patients and controls. The phyloseq object is then used as input for the main analysis. Similar to the aforementioned tests we included age, sex, BMI, smoking status, number of teeth and SBI as covariates [66, 67]. We calculated 95% confidence intervals for the estimated log-fold-changes using the critical t-values derived from the student's t-distribution with degrees of freedom approximated as the sample size minus the number of model parameters (42 for salivary and 43 for supragingival samples). Specifically, critical values were obtained in R using the qt() function with the basic command 'qt(0.975, df = 42)', corresponding to the 97.5th percentile of the t-distribution (see Supplementary scripts). The graphical processing of the ANCOM-BC2 results was also carried out in Python using the modules pandas (version 2.0.0) and matplotlib. For the visualization, we only included taxa which in addition to being significantly different after multiple testing correction also did not show any association to the relevant covariates number of teeth and SBI.

As the third method, absolute read counts were analyzed in R using Limma with the limma (version 3.58.1) and edgeR libraries (version 4.0.16). Originally developed for gene expression analysis in microarray and RNA-seq experiments, Limma employs linear modeling to assess differential abundance patterns and is particularly suitable for designs involving multiple covariates. One of the model assumptions of Limma is homogeneity of variance which can be disturbed by outliers or taxa with extremely low expression. A common way to deal with this problem is the strict filtering of noisy data. We therefore first filtered the raw counts by removing taxa with less than ten reads in more than 70% of the samples. This cut-off was chosen based on empirical inspection of the mean–variance trend plot after voom transformation to increase model stability: This plot allows the visual assessment of the relation between mean taxa abundance and variance, effectively enabling the testing of different cut-offs increasing model stability while still including as many taxa as possible. This filtering process aims to balance the trade-off between maximizing the signal-to-noise ratio, by removing low-abundance noisy taxa, and minimizing the loss of biologically relevant information, thereby preserving statistical power for downstream analyses. The read counts were then normalized via Trimmed Mean of M-values with Singleton Pairing (TMMwsp) and the log-based voom transformation was performed. The group was incorporated as the main binary outcome of interest and age, sex, BMI, smoking status, number of teeth and SBI as covariates. The paired design was explicitly included using the function 'duplicateCorrelation' with a blocking factor representing the subject pairing. The final model was fit using the function 'lmFit' with the estimated correlation and empirical Bayes shrinkage of standard errors was performed via 'eBayes' to improve detection power (see Supplementary scripts) [68–71].

As the fourth and last method for differential abundance analysis, we used the ALDEx2 (version 1.34.0) library in R. We first filtered the raw counts the same way we did with Limma, as ALDEx2 does not include default filtering criteria. ALDEx2 models the observed counts as probabilities by Monte Carlo sampling from the Dirichlet distribution, generating for each feature a vector of *n* instances (default* n* = 128). This vector of probabilities is then center log-ratio transformed and significance testing followed by multiple testing correction is performed using the 'paired.test = TRUE' option for paired testing and the 'denom = “lvha”' option (low variance, high abundance) to further increase the focus on more robust taxa. Only paired samples were included in this analysis. Therefore, patient02 and control26 in case of salivary samples, and only patient02 for supragingival samples were left out (see Supplementary scripts) [72].

#### Cross-validation

To further increase the robustness of our analysis we repeated all statistical tests incorporating covariates with the addition of the education years as another covariate to see if taxa remained significantly different (see Discussion and Supplementary Table 13–16). We also again used GLMMMiRKAT the same way as before basically switching the binary group condition as the outcome with the education years and incorporating the group as a covariate to identify associations between the microbial data and the education years (see Discussion and Supplementary Table 12).

As another cross-validation we considered a more integrative way of using the combined results of the different sequencing platforms and modeling them as batch-effects. For this purpose, we combined the result tables with raw read counts of the three salivary datasets and first filtered them the same way we did for the analysis with Limma and ALDEx2. Next, we used these combined data as input for ANCOM-BC2 and Limma, similar to the aforementioned workflows yet with some distinct differences: First and most important the sequencing method was incorporated as an additional covariate and only taxa not showing a batch-effect were considered as robust results. Also, the threshold for adjusted P values was lowered to 0.01 to account for the artificially increased sample number. Additionally, we changed the option 'neg_lb' to 'TRUE' as the sample number now surpassed *n* = 30, mentioned by the authors as a useful threshold to change this parameter. The Limma workflow was primarily changed by adding the method as an additional covariate. ALDEx2 was not used for this cross-validation as it does not have any option to include the method as a covariate. We then lastly identified the taxa identified by both methods and incorporated this as an additional robustness indicator in our Core results (see Tables 2, 3 and Supplementary Table 11).

As a third cross-validation method we evaluated to which degree our results are driven by the pairing of our samples. To achieve this, we repeated the analysis with GLMMMiRKAT, as well as Limma and ALDEx2 with a permutation approach randomly changing the pairing of patients and controls while keeping the basic group comparisons across 1000 permutations. For more efficient parallelization, the future (version 1.67.0) and future.apply libraries (version 1.20.0) were additionally used (see Supplementary scripts). For ALDEx2 and Limma the mean results of the 1000 permutations were calculated with custom python-scripts.

#### Comparison of sequencing methods

In addition to comparing patients and controls, the three sequencing approaches were examined across all taxonomic levels in R. To this end, we first concatenated the relative abundance tables from the three sequencing methods to create a unified dataset for the subsequent analyses. Next, pairwise Mantel-tests between sequencing methods were performed using the vegan library. The Mantel-test evaluates the correlation between two dissimilarity matrices, traditionally using the Pearson-correlation, although modern implementations also support Spearman-correlation. Because the assumption of normality underlying the Pearson-correlation is rarely met in relative abundance data, while the Spearman-correlation is purely rank-based, we applied both correlation types using again Bray–Curtis-distances as the dissimilarity measure. Significance was determined via permutation testing, in which the distance matrices are repeatedly randomized to compare the mean correlation to the observed correlation [73]. To account for multiple testing, P values were subsequently adjusted using again the Benjamini–Hochberg correction. Following this analysis, we assessed the overlap in taxa detected by the different sequencing methods and visualized these intersections using Venn-diagrams generated with the VennDiagram (version 1.7.3) and grid (version 4.5.1) libraries. To evaluate the robustness of the workflow, we repeated all analyses using two alternative filtering criteria: (i) retaining taxa with a minimum mean relative abundance of 0.1% across the dataset and (ii) retaining taxa present in at least 30% of the samples for each of the three sequencing methods (in both cases with remaining taxa summed as 'Others') (see Supplementary scripts).

#### Remaining statistical operations

Remaining between-group differences were assessed using the T-test for dependent samples for normally distributed continuous variables, the Wilcoxon-signed-rank-test for non-normally distributed continuous variables and the χ^2^-test and Fisher's-exact-test for categorical variables. Accordingly, patient02 was again left out from these analyses to only include paired samples. Shapiro-Wilks-test and Levene-test were used to test for normal distribution and equality of variance. Pairwise comparisons were performed using two-sided tests. A P value of less than 0.05 was considered statistically significant. For inferential statistics, results are presented as P values only for the Wilcoxon-signed-rank-test and with 95% confidence intervals for the T-test. Analyses were performed in R using the car library. Barplots and heatmaps were produced in R using the dplyr, grid, tidyr (version 1.3.1), stringr (1.5.2), ComplexHeatmap (version 2.25.2), circlize (version 0.4.16) and RcolorBrewer (version 1.1.3) libraries. R-scripts with detailed statistical workflows are provided as supplementary material.

## Results

### Study cohort and oral status

A total of 51 subjects were recruited, of whom 26 were diagnosed with AD. Age, sex distribution, weight, height, BMI, education years, MMSE, number of teeth and SBI are depicted in detail in Table 1. As the number of teeth of three patients and three control subjects was too low to perform the SBI, the index was analyzed in two ways: As a continuous variable without the missing subjects tested via Wilcoxon-signed-rank-test and as a categorical variable via Fisher's-exact-test with the missing subjects defined as a fifth 'missing' stage. This way, the SBI could also be incorporated as a covariate for β-diversity analyses.

**Table 1:** General subject characteristics

Parameters	Patients (mean ± standard deviation) (*n* = 26)	Controls (mean ± standard deviation) (*n* = 25)	*P* value	95% CI
Age (years)	67.85 (± 8.26)	67.36 (± 9.15)	0.85	−2.88, 2.40
Sex (n female (%))	14 (53.8)	13 (52)	1	-
Weight (kg)	69.50 (± 13.04)	77.42 (± 12.64)	0.06	−0.28, 15.12
Height (m)	1.71 (± 0.10)	1.73 (± 0.09)	0.52	−0.04, 0.08
BMI (kg/m^2)	23.73 (± 3.52)	25.79 (± 3.91)	0.02*	0.31, 3.74
Education years (years)	15.31 (± 3.94)	17.18 (± 2.98)	0.02*	-
MMSE (points)	23.77 (± 3.80)	29.48 (± 1.12)	< 0.01*	-
Number of teeth (FDI)	23.42 (± 7.49)	24.20 (± 6.85)	0.50	-
SBI (stage 1–4)	1.48 (± 3.59)	1.55 (0.67)	1	-
SBI (stage 1–4, or missing)	-	-	1	-
Smoking status	-	-	1	-

Parameters were compared using paired T-test for dependent samples, respectively Wilcoxon-signed-rank-test, respectively χ^2^-test (Sex) or Fisher’s exact test (SBI including missing and Smoking status). Means ± standard deviation are shown. Statistically significant differences (*p* < 0.05) are indicated by *

The two groups only showed a significant difference in BMI (*p* = 0.02; 95% CI: 0.31, 3.74), education years (*p* = 0.02) and MMSE (*p* < 0.01) with all three measures being higher in controls. No significant differences were found between the two groups regarding oral health status and dental and oral health history (see Supplementary Table 1).

When testing for associations between the microbial data and the number of teeth, SBI and the smoking status with GLMMMiRKAT across the different phylogenetic levels only one significant association between the full-length sequencing of supragingival samples on the species-level and the number of teeth was found (Bray–Curtis: *p* < 0.05 (0.047); Omnibus: *p* = 0.05). No significant associations between BMI and the microbial data were identified (see Supplementary Table 12). Most importantly, no significant differences were found between the two groups regarding oral health status and dental and oral health history (see Supplementary Table 1).

### Analysis of saliva samples

Salivary samples were sequenced using three different methods: Metagenomic and 16S full-length sequencing with Oxford Nanopore technology and 16S short-read sequencing of the variable regions V3 and V4 on a NextSeq2000 Illumina platform. Patient26 was unable to collect enough saliva, therefore a total of 50 salivary samples were analyzed.

**Fig. 2:**
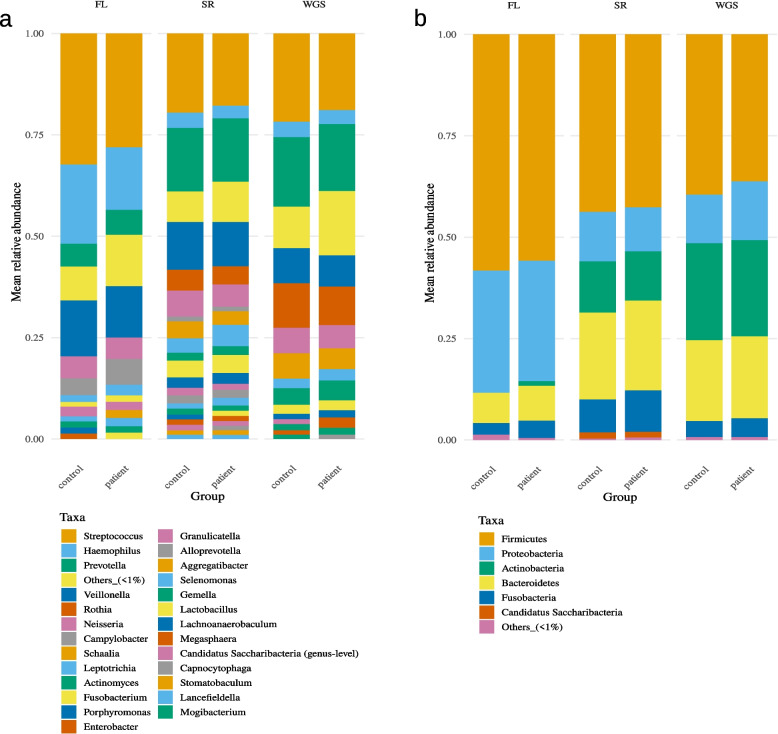
Mean relative abundances of the salivary samples for the genus- (**a**) and phylum-level (**b**). The columns represent the 16S full-length (FL), 16S short-read (SR) and metagenomic (WGS) sequencing results divided by patients and controls. Taxa with a mean abundance below 1% are summed as Others_1%. Abbreviations: FL, full-length; SR, short-read; WGS, whole-genome-sequencing

Intra-partnership correlation was significantly higher, and Bray–Curtis-distance significantly lower for all three sequencing methods for at least the species- and genus-level, regardless of correlation type and filtering of the data. This difference is in part lost at higher phylogenetic levels (see Supplementary Table 4). An overview of the mean relative abundances for the genus- and phylum-level is presented in Fig. 2.

#### Metagenomic sequencing

In total, the metagenomic sequencing of 50 salivary samples generated 11,578,948 reads with an average length of 2,487 bp and an average read count of 231,578. Of these reads, a mean of 84.98% was identified as human reads and consequently removed. The remaining reads had an average length of 2,037 bp, an average read count of 35,099 and a median read count of 1,866 reads.

**Fig. 3:**
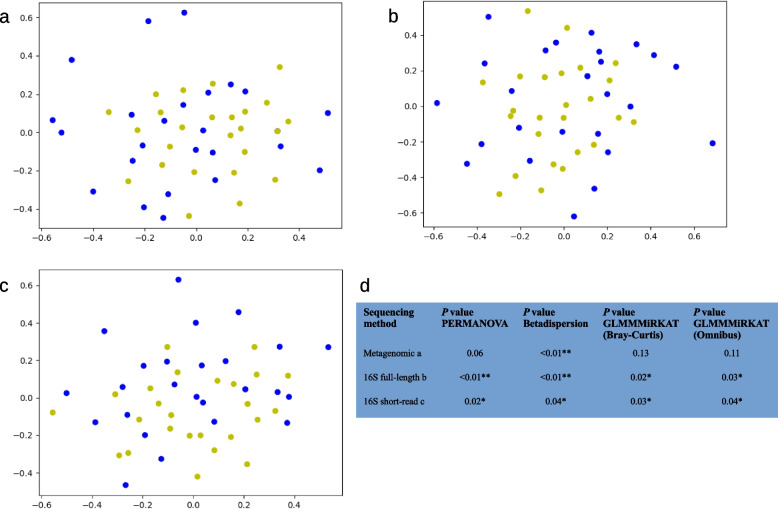
Bray–Curtis based PCoAs of metagenomic (**a**), 16S full-length (**b**), and 16S short-read (**c**) sequencing of salivary samples. Patients are shown in blue, and controls in yellow. The samples from the control group show a tighter grouping across all three methods while the samples from the patient group are more widely dispersed. (**d**) *P* values for PERMANOVA, Betadispersion, as well as GLMMMiRKAT (Bray–Curtis and Omnibus) testing are presented in the table. Statistically significant differences (*p* < 0.05) are indicated by *. Abbreviations: PCoA, Principal Coordinate Analysis; PERMANOVA, Permutational Multivariate Analysis Of Variance; GLMMMiRKAT, Generalized Linear Model Microbiome Regression-based Kernel Association Test

For both statistical approaches and both α-diversity indices, there were no significant differences between patients and controls (see Supplementary Table 5). To determine β-diversity, Bray–Curtis-dissimilarity and PCoA were calculated. When analyzing the metagenomic sequencing of 50 salivary samples, a total of 2,122 different species were identified. Figure 3a shows the distribution of patients (in blue) and controls (in yellow). Although the samples from both groups overlap, the samples from the control group show a narrower grouping while the samples from the patient group are more widely dispersed. This visual result is supported by PERMANOVA and Betadispersion testing showing no significant difference in centroids (*p* = 0.06) but in dispersion (*p* < 0.01). None of the covariates were significant in PERMANOVA testing (see Fig. 3d and Supplementary Table 6). Concordant with PERMANOVA testing with GLMMMiRKAT also found no significant association between species-level metagenomic data and group status (see Fig. 3d and Supplementary Table 7). Comparing the original GLMMMiRKAT results to the mean results after randomly changing the sample pairing across 1000 permutations the original results show consistently lower P values across all taxonomic levels (see Supplementary Table 7).

**Fig. 4:**
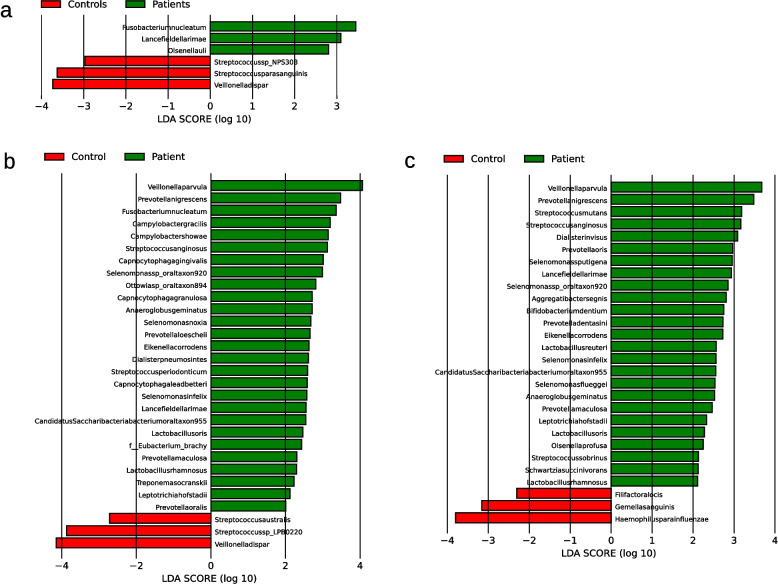
Bacterial species identified as statistically significant by LEfSe in the metagenomic (**a**), 16S full-length (**b**), and 16S short-read (**c**) sequencing analysis of salivary samples. Taxa that have achieved an LDA score of at least 2 or −2 in the linear discriminant analysis are shown. Species with a higher abundance in the patient group are shown in green and for the control group in red. Abbreviations: LEfSe, Linear Discriminant Analysis Effect Size; LDA, Linear Discriminant Analysis

With LEfSe six species were identified whose relative abundance differed significantly between patients and controls. In descending order of LDA score, *Fusobacterium nucleatum, Lancefieldella rimae* and *Olsenella uli* were identified as species with higher abundance in the patient group and *Veillonella dispar, Streptococcus parasanguinis* and *Streptococcus sp. NPS 308* as species with higher abundance in the control group (see Fig. 4a).

**Fig. 5:**
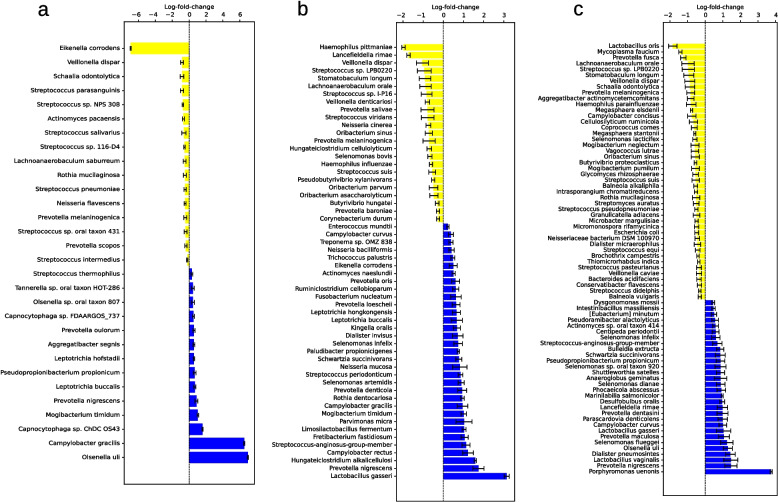
Bacterial species identified as statistically significant by ANCOM-BC2 and without association to number of teeth or SBI in the metagenomic (**a**), 16S full-length (**b**) and 16S short-read (**c**) sequencing analysis of salivary samples. Bars with whiskers represent log-fold-change with standard error. Taxa with a higher abundance in the patient group are shown in blue and for the control group in yellow. Abbreviations: ANCOM-BC2, Analysis of Compositions of Microbiomes with Bias Correction 2; SBI, Sulcus bleeding Index

Using ANCOM-BC2, a total of 65 bacterial species were identified whose absolute read count showed a statistically significant difference between the patient and control groups. Of these, 30 species did not exhibit significant associations with either SBI or the number of teeth with 16 species with a higher abundance in the patient and 14 with a higher abundance in the control group (see Fig. 5a). Also 19 genera were identified (see Supplementary Table 9). Besides others, species of particular interest include *Olsenella uli*, *Prevotella nigrescens* and *Leptotrichia hofstadii* in patients and *Veillonella dispar*, *Streptococcus parasanguinis* and *Prevotella melaninogenica* in controls.

With Limma only the two species *Prevotella nigrescens* and *Streptococcus sp. oral taxon 431* were identified (see Supplementary Table 8) while ALDEx2 did not identify any species as significantly different in the metagenomic data. Similar to the GLMMMiRKAT analysis, the species identified by Limma had higher P values in the permutational analysis as well as wider confidence intervals (see Supplementary Table 8).

If not mentioned otherwise, the same trend was observed across all subsequent permutational analyses with GLMMMiRKAT, Limma and ALDEx2. For detailed results see Supplementary Table 7, 8 and 10.

#### 16S full-length sequencing

In total, the 16S full-length sequencing of 50 salivary samples generated 5,611,792 reads with an average length of 1,400 bp and an average read count of 112,236. For α-diversity no significant difference was identified for Simpson index, yet the Shannon index was significantly higher in patients (*p* = 0.04) (see Supplementary Table 5).

When analyzing the full-length 16S sequencing of 50 salivary samples, a total of 511 different species were identified. The Bray–Curtis based PCoA shows a less clear separation between patients (in blue) and controls (in yellow) than the metagenomic sequencing samples (see Fig. 3b). Nevertheless, a further dispersion of the samples from the patient group can be recognized which is supported by a significant difference in centroids and dispersion identified via PERMANOVA (*p* < 0.01) and Betadispersion-test (*p* < 0.01). Again, none of the covariates were significant in PERMANOVA testing (see Fig. 3d and Supplementary Table 6). GLMMMiRKAT also found a significant association between microbial data and group status (Bray–Curtis: *p* = 0.02; Omnibus: *p* = 0.03) (see Fig. 3d and Supplementary Table 7).

Thirty species were identified with LEfSe with 27 in patients and three in controls including among others *Prevotella nigrescens*, *Streptococcus anginosus*, *Anaeroglobus geminatus*, *Prevotella maculosa* and *Leptotrichia hofstadii* in patients and *Veillonella dispar* in controls (see Fig. 4b).

Using ANCOM-BC2, a total of 87 bacterial species were identified whose absolute read count showed a statistically significant difference between the patient and control groups. Of these, 56 species did not exhibit significant associations with either SBI or the number of teeth with 33 species with a higher abundance in the patient and 23 with a higher abundance in the control group (see Fig. 5b). Also 35 genera were identified (see Supplementary Table 9). Besides others, species of particular interest included *Prevotella nigrescens* and *Selenomonas infelix* in patients as well as *Prevotella melaninogenica*, *Streptococcus sp. LPB0220* and *Veillonella dispar* in controls.

With Limma 18 species were identified including *Prevotella maculosa*, *Streptococcus anginosus*, *Anaeroglobus geminatus* and *Prevotella nigrescens* in patients and *Veillonella dispar*, *Streptococcus parasanguinis* and *Streptococcus sp*. *LPB0220* in controls (see Supplementary Table 8). ALDEx2 identified ten species exclusively in patients, including *Anaeroglobus geminatus*, *Leptotrichia hofstadii*, *Prevotella maculosa* and *Prevotella nigrescens* (see Supplementary Table 10).

#### 16S short-read sequencing

In total, short-read 16S sequencing of 50 salivary samples generated 105,339,270 reads with an average length of 561 bp and an average read count of 2,106,785.

There was no significant difference between patients and controls for the Simpson index. However, there was again a significantly higher result for the Shannon index in the patient group (*p* = 0.02; 95% CI: 0.03, 0.33) (see Supplementary Table 5).

The Bray–Curtis based PCoA shows a separation between patients (in blue) and controls (in yellow), comparable to the salivary samples of the metagenomic sequencing (see Fig. 3c). As with the full-length data, PERMANOVA (*p* = 0.02) as well as Betadispersion-test (*p* = 0.04) confirmed a significant difference in centroids as well as dispersion. Again, none of the covariates was significant in PERMANOVA testing (see Fig. 3d and Supplementary Table 6). GLMMMiRKAT also found a significant association between microbial data and group status (Bray–Curtis: *p* = 0.03; Omnibus: *p* = 0.04) (see Fig. 3d and Supplementary Table 7).

28 species were identified with LEfSe with 25 in patients and three in controls including *Prevotella nigrescens*, *Streptococcus anginosus* and *Anaeroglobus geminatus* (see Fig. 4c). Using ANCOM-BC2, a total of 115 bacterial species were identified whose absolute read count showed a statistically significant difference between the patient and control groups. Of these, 74 species did not exhibit significant associations with either SBI or the number of teeth with 30 species with a higher abundance in the patient and 44 with a higher abundance in the control group (see Fig. 5c). Also 56 genera were identified (see Supplementary Table 9). Besides others, species of particular interest included *Prevotella nigrescens*, *Olsenella uli* and *Prevotella maculosa* in patients and *Prevotella melaninogenica*, *Veillonella dispar* and *Streptococcus sp*. *LPB0220* in controls.

With Limma 17 species were identified including *Streptococcus anginosus*, *Prevotella nigrescens* and *Prevotella maculosa* in patients and *Steptococcus parasanguinis* in controls (see Supplementary Table 8). Also, six species were identified with ALDEx2 again only in patients and including *Anaeroglobus geminatus*, *Prevotella maculosa* and *Prevotella nigrescens* (see Supplementary Table 10).

### Analysis of supragingival samples

In contrast to the salivary samples, due to a lower mean DNA yield, the supragingival samples were sequenced only in two ways: A 16S full-length sequencing with Oxford Nanopore technology and a 16S short-read sequencing on a NextSeq2000 Illumina platform.

**Fig. 6:**
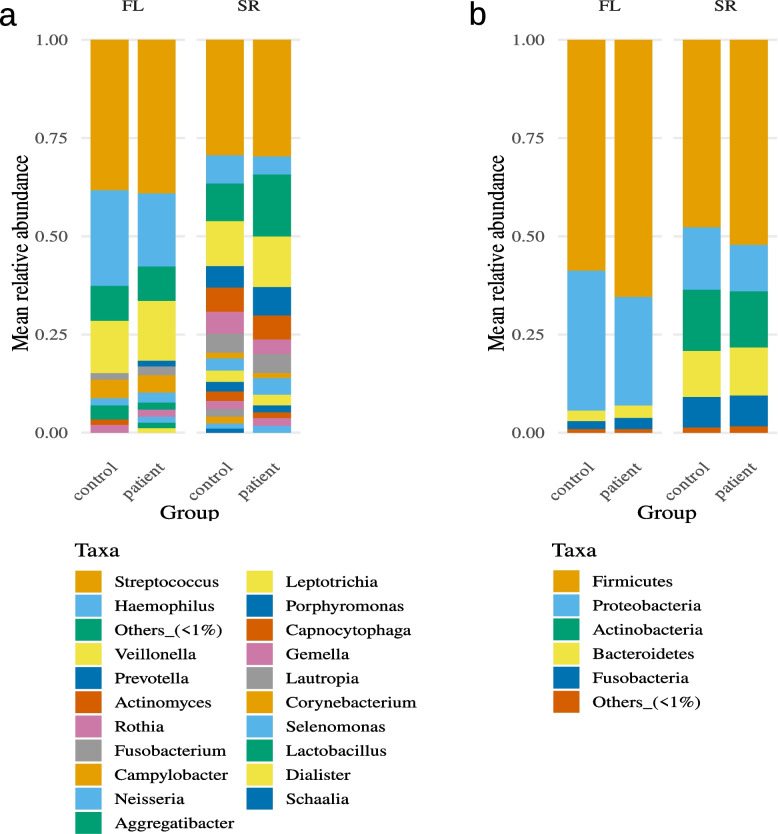
Mean relative abundances of the supragingival samples for the genus- (**a**) and phylum-level (**b**). The columns represent the 16S full-length (FL) and 16S short-read (SR) sequencing results divided by patients and controls. Taxa with a mean abundance below 1% are summed as Others_1%. Abbreviations: FL, full-length; SR, short-read

Intra-partnership correlation was significantly higher, and Bray–Curtis-distance significantly lower for both sequencing methods for at least the species-level, regardless of correlation type and filtering of the data. However, for these samples the difference is in part already lost at the genus level (see Supplementary Table 4). An overview of the mean relative abundances for the genus- and phylum-level is presented in Fig. 6.

#### 16S full-length sequencing

In total, 3,414,757 reads with an average length of 1,375 bp and an average read count of 66,956 were sequenced from 51 supragingival samples using full-length 16S sequencing.

The α-diversity (Simpson and Shannon index) showed no significant difference between patients and controls for either index (see Supplementary Table 5).

**Fig. 7:**
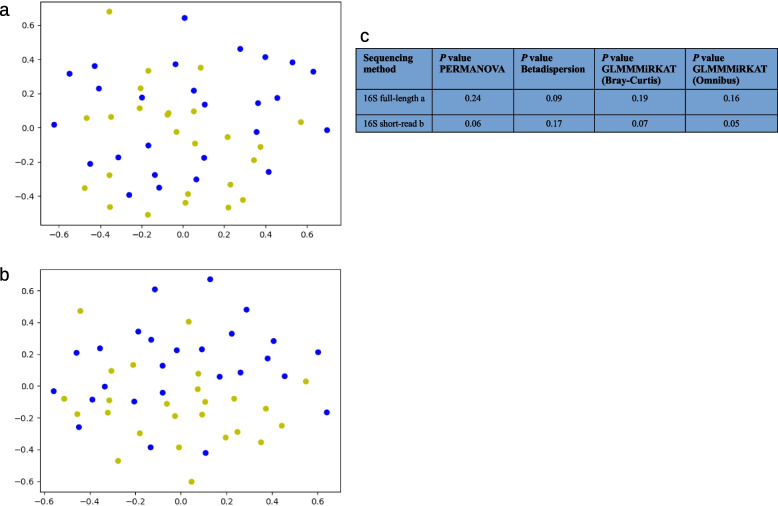
Bray–Curtis based PCoAs of 16S full-length (**a**), and 16S short-read (**b**) sequencing of supragingival samples. Patients are shown in blue, and controls in yellow. For the 16S full-length sequencing the samples from the control group show a slightly tighter grouping while the samples from the patient group are more widely dispersed. For the 16S short-read sequencing the two groups show a comparable distribution but a clearer separation of the two groups. (**c**) *P* values for PERMANOVA, Betadispersion, as well as GLMMMiRKAT testing (Bray–Curtis and Omnibus) are presented in the table. Statistically significant differences (*p* < 0.05) are indicated by *. Abbreviations: PCoA, Principal Coordinate Analysis; PERMANOVA, Permutational Multivariate Analysis Of Variance; GLMMMiRKAT, Generalized Linear Model Microbiome Regression-based Kernel Association Test

The Bray–Curtis based PCoA shows a less clear separation between patients (in blue) and controls (in yellow) than the salivary samples of the metagenomic sequencing (see Fig. 7a). Nevertheless, a further dispersion of the samples from the patient group can be recognized. However, PERMANOVA, Betadispersion and GLMMMiRKAT showed no significant effect for the group status yet instead in case of PERMANOVA for the number of teeth (*p* = 0.03) confirming the earlier results from GLMMMiRKAT in "Study cohort and oral status" section (see Fig. 7c and Supplementary Table 6 and 7).

**Fig. 8:**
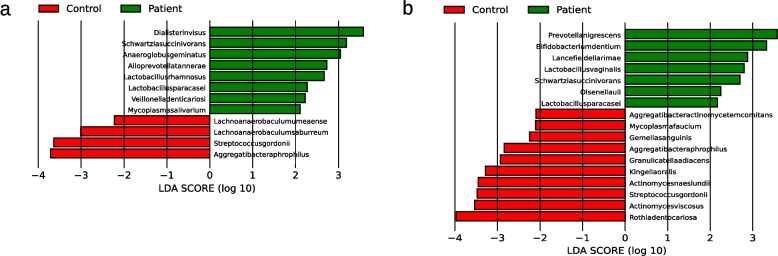
Bacterial species identified as statistically significant by LEfSe in the 16S full-length (**a**), and 16S short-read (**b**) sequencing analysis of supragingival samples. Taxa that have achieved an LDA score of at least 2 or −2 in the linear discriminant analysis are shown. Species with a higher abundance in the patient group are shown in green and for the control group in red. Abbreviations: LEfSe, Linear Discriminant Analysis Effect Size; LDA, Linear Discriminant Analysis

**Fig. 9:**
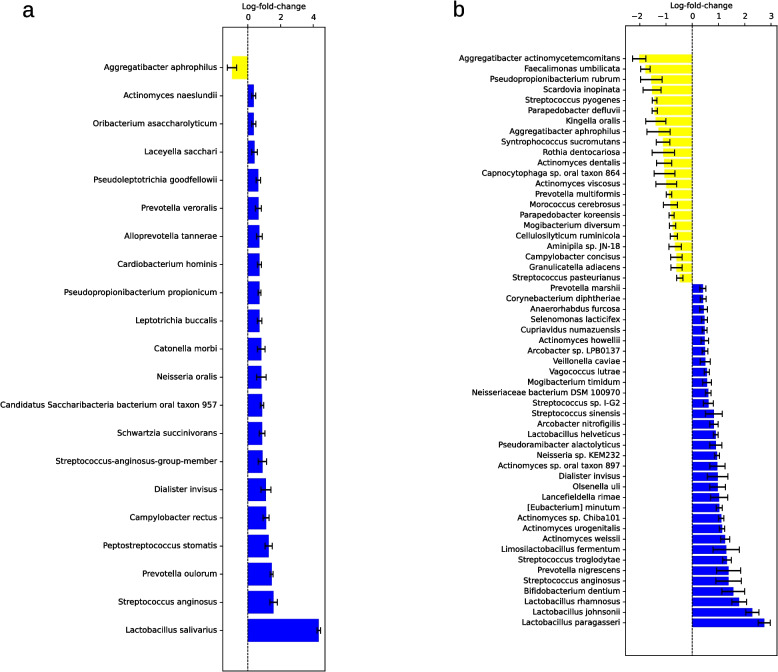
Bacterial species identified as statistically significant by ANCOM-BC2 and without association to number of teeth or SBI in the 16S full-length (**a**) and 16S short-read (**b**) sequencing analysis of supragingival samples. Bars with whiskers represent log-fold-change with standard error. Taxa with a higher abundance in the patient group are shown in blue and for the control group in yellow. Abbreviations: ANCOM-BC2, Analysis of Compositions of Microbiomes with Bias Correction 2; SBI, Sulcus bleeding Index

Twelve species were identified with LEfSe, eight in patients and four in controls (see Fig. 8a). Using ANCOM-BC2, a total of 46 bacterial species were identified whose absolute read count showed a statistically significant difference between the patient and control groups. Of these, 21 species did not exhibit significant associations with either SBI or the number of teeth with 20 species with a higher abundance in the patient and one, namely *Aggregatibacter aphrophilus*, with a higher abundance in the control group (see Fig. 9a). Also 11 genera were identified (see Supplementary Table 9). Neither Limma nor ALDEX2 identified any significant difference.

#### 16S short-read sequencing

In total, the short-read 16S sequencing of 51 supragingival samples generated 96,823,518 reads with an average length of 562 bp and an average read count of 1,898,500.

As seen for the 16S full-length sequencing, no significant difference between patients and controls for Shannon and Simpson indices was found (see Supplementary Table 5).

The Bray–Curtis based PCoA shows a separation between patients (in blue) and controls (in yellow). This separation is more pronounced than in the salivary and supragingival samples of the full-length 16S sequencing, as well as the salivary samples of the short-read 16S sequencing (see Fig. 7b). However, neither PERMANOVA nor Betadispersion-test as well as GLMMMiRKAT produced significant results including covariate testing in case of PERMANOVA (see Fig. 7c and Supplementary Table 6 and 7).

19 species were identified with LEfSe, seven in patients and 12 in controls including *Prevotella nigrescens* and *Olsenella uli* in patients (see Fig. 8b). Also, 88 species were identified with ANCOM-BC2, of whom 55 did not exhibit significant associations with either SBI or the number of teeth including 33 species in patients and 22 in controls (see Fig. 9b). Also 33 genera were identified (see Supplementary Table 9). In case of Limma only *Bifidobacterium dentium* was identified as being of higher abundance in patients (see Supplementary Table 8) while ALDEx2 again found no significant differences.

### Comparison of sequencing methods

#### Saliva

All three sequencing methods positively and significantly correlated with each other across all taxonomic levels and regardless of the correlation method (Pearson or Spearman). The highest correlations were thereby identified for the species-level, followed by genus and then decreasing towards the higher phylogenetic levels. These correlations dropped only marginally when filtering by mean abundance, respectively prevalence with the species-level showing the biggest changes yet still keeping the highest correlations. However, also consistently through all taxonomic levels, correlation methods and filtering steps, metagenomic and 16S short-read sequencing data showed the highest correlation (species_Pearson: Mantel_r = 0.78, p_adj < 0.01; genus_Pearson: Mantel_r = 0.71, p_adj < 0.01), followed by 16S full-length sequencing and 16S short-read sequencing (species_Pearson: Mantel_r = 0.74, p_adj < 0.01; genus_Pearson: Mantel_r = 0.56, p_adj < 0.01) and lastly metagenomic and 16S full-length sequencing (species_Pearson: Mantel_r = 0.71, p_adj < 0.01; genus_Pearson: Mantel_r = 0.52, p_adj < 0.01) (see Supplementary Table 17).

**Fig. 10:**
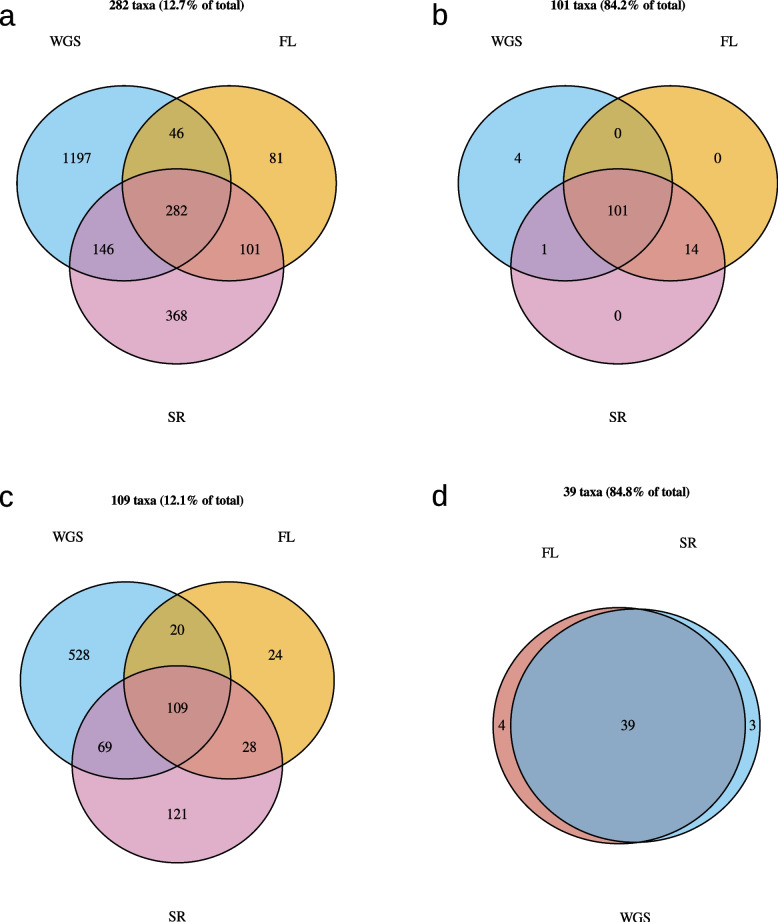
Venn-diagrams showing the overlap in taxon identification for the salivary samples between the results of the metagenomic (WGS), 16S full-length (FL) and 16S short-read (SR) sequencing. Presented are the results for different taxonomic levels and filtering approaches: A and b show the species-level with the overlap of the unfiltered dataset (**a**) and the dataset filtered by a minimum relative abundance of 0.1% (**b**). C and d show the equivalent results for the genus-level. Abbreviations: FL, full-length; SR, short-read; WGS, whole-genome-sequencing

When looking at the different taxa identified with each method, the unfiltered data show the smallest overlap for the species- and genus-level with only 12.7% and 12.1% of all identified taxa identified with all three methods. This degree of overlap increases for the higher phylogenetic levels but still only reaches 42.3% at the phylum-level. However, when looking at the data filtered by minimum abundance, the overlap increases to a minimum of 84.2% at the species- and 84.8% at the genus-level (see Fig. 10 and Supplementary Table 18).

#### Supragingival

Similar to the salivary results, a positive and significant correlation between the 16S full-length and 16S short-read data was found across all taxonomic levels and for Pearson-, as well as Spearman-correlation. This correlation was again highest for the species-level (Pearson: Mantel_r = 0.73, p_adj < 0.01) and only slightly lower for the genus-level (Pearson: Mantel_r = 0.71, p_adj < 0.01) to then decrease stepwise towards the higher phylogenetic levels. These correlations again only changed marginally when filtering by mean abundance (species_abun_Pearson: Mantel_r = 0.73, p_adj < 0.01; genus_abun_Pearson: Mantel_r = 0.71, p_adj < 0.01) or prevalence (species_prev_Pearson: Mantel_r = 0.69, p_adj < 0.01; genus_prev_Pearson: Mantel_r = 0.70, p_adj < 0.01) (see Supplementary Table 17).

**Fig. 11:**
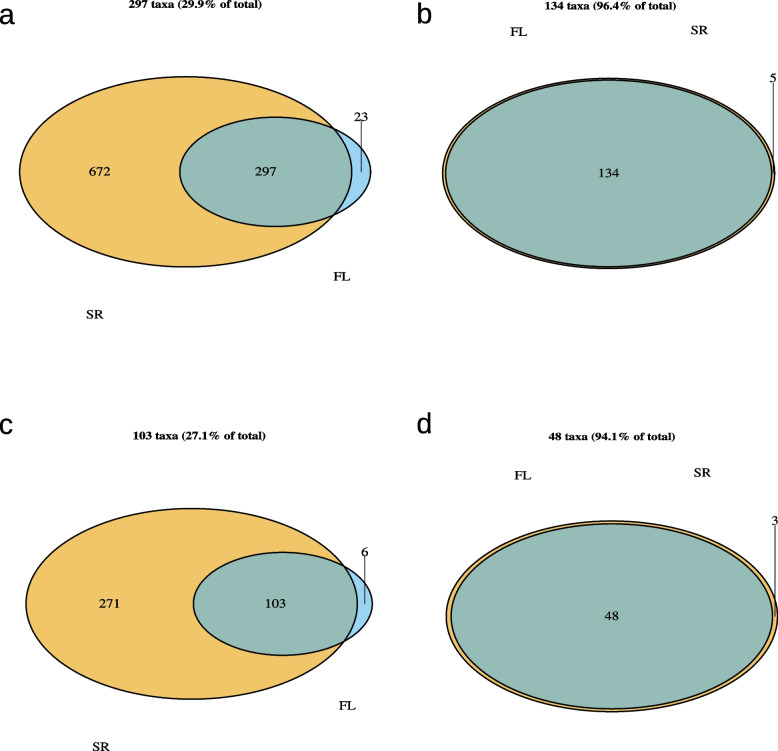
Venn-diagrams showing the overlap in taxon identification for the supragingival samples between the results of the 16S full-length (FL) and 16S short-read (SR) sequencing. Presented are the results for different taxonomic levels and filtering approaches: A and b show the species-level with the overlap of the unfiltered dataset (**a**) and the dataset filtered by a minimum relative abundance of 0.1% (**b**). C and d show the equivalent results for the genus-level. Abbreviations: FL, full-length; SR, short-read

As with the salivary samples, the unfiltered supragingival datasets had the smallest overlap with 29.9% of all identified species and 27.1% of all identified genera. However, this again changed after filtering by mean abundance with 96.4% and 94.1% overlap for the species- and genus-level (see Fig. 11 and Supplementary Table 18).

### Core dysbiosis

To narrow down the most robust results across the different sequencing methods and statistical approaches we searched for taxa which were identified multiple times as significantly different in a consistent manner. In this sense a taxon was considered as part of what we call the 'Core dysbiosis' if it was (i) identified at least three times with either ANCOM-BC2, Limma or ALDEx2 without necessarily being identified with all three methods, (ii) the identification shows a consistent trend towards patients or controls and (iii) the taxon was identified with at least two of the three sequencing methods. In addition, the identification with LEfSe, the integrated sensitivity analysis by changing pseudocounts with ANCOM-BC2 and the identification with education years as an additional covariate were used as sensitivity analyses for the taxa of the Core dysbiosis, assuming that the most robust differences should also be recognized with these additional analyses.

**Fig. 12:**
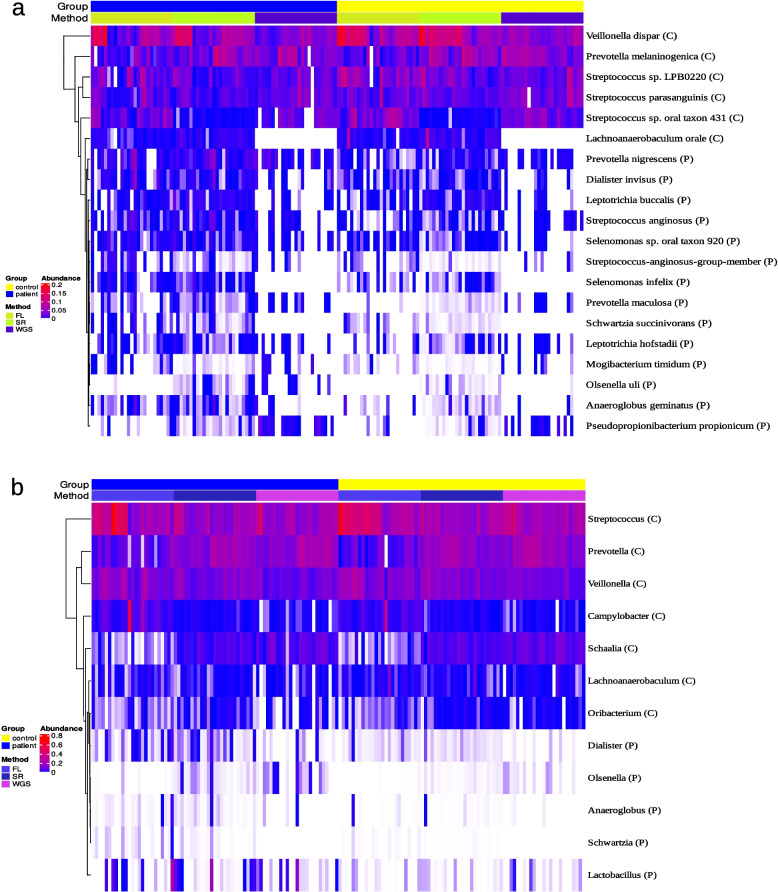
Heatmaps visualizing the relative abundances of the species (**a**) and genera (**b**) of the Core dysbiosis in the salivary samples divided by patients and controls, as well as sequencing methods. Taxa with higher abundances in patients, respectively controls are marked with a P and C. Abbreviations: FL, full-length; SR, short-read; WGS, whole-genome-sequencing

**Fig. 13:**
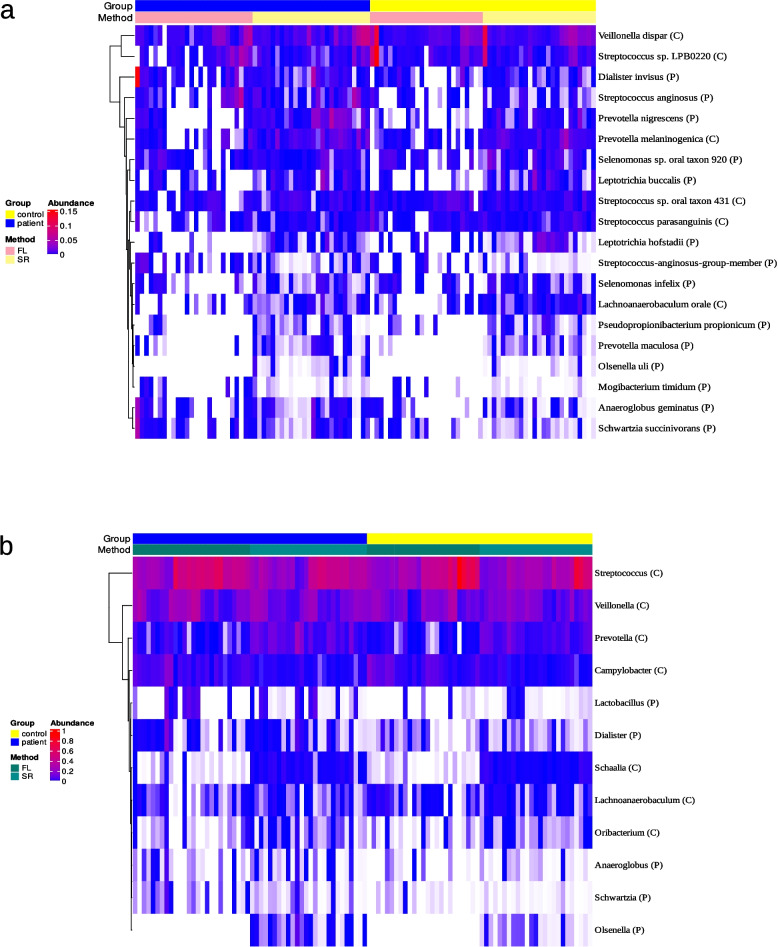
Heatmaps visualizing the relative abundances of the species (**a**) and genera (**b**) of the Core dysbiosis in the supragingival samples divided by patients and controls, as well as sequencing methods. Taxa with higher abundances in patients, respectively controls are marked with a P and C. Abbreviations: FL, full-length; SR, short-read

Across all results we identified 20 species and 12 genera which were identified at least three times with at least two sequencing methods. Of these, six species and nine genera were predominantly increased in controls with the rest showing higher abundances in patients (see Tables 2 and 3 and Figs. 12 and 13). For the species, most identifications were confirmed when repeating the analysis with inclusion of education years as additional covariate. In addition, most taxa passed the sensitivity analysis with pseudo-counts in the analysis with ANCOM-BC2. However, less than half of the identifications were in concordance with the results from LEfSe (see Table 2). Similarly, most of the genera were not identified with LEfSe and lost after inclusion of education years. However, again the majority of the taxa identified with ANCOM-BC2 passed the sensitivity analysis (see Table 3).

**Table 2:** Total intersection of all bacterial species identified using ANCOM-BC2, Limma and ALDEx2 in salivary and supragingival samples from metagenomic, full-length and 16S short-read sequencing. The material, sequencing method, differential abundance analysis tool, log-fold-change with SE and 95% CI, respectively diff.btw with effect size and 95% CI for ALDEx2 and group with higher occurrence are shown. Also, the identification with LEfSe, the passing of the internal sensitivity analysis with pseudo-counts by ANCOM-BC2 and the passing of the cross-validation with inclusion of education years as additional covariate are shown. Abbreviations: ALDEx2, ANOVA-Like Differential Expression 2; ANCOM-BC2, Analysis of Compositions of Microbiomes with Bias Correction 2; CI, Confidence interval; LefSe, Linear Discriminant Analysis Effect Size; Limma, Linear Models for Microarray; SE, Standard error

Taxon	Material	Sequencing method	DA method	Log-fold-change (± SE; 95% CI)	diff.btw (effect; 95% CI)	Group	Identified with LefSe		Passed sensitivity analysis	Identified with education years as covariate
Prevotella nigrescens	Saliva	WGS	limma	1.60 (± 0.51; 0.58, 2.62)		Patient	WGS_sal	FALSE		TRUE
Prevotella nigrescens	Saliva	FL	limma	2.43 (± 0.76; 0.89, 3.97)		Patient	FL_sal	TRUE		TRUE
Prevotella nigrescens	Saliva	SR	limma	2.18 (± 0.32; 1.53, 2.83)		Patient	SR_sal	TRUE		TRUE
Prevotella nigrescens	Saliva	FL	ALDEX		2.78 (0.63; −2.78, 3.26)	Patient				TRUE
Prevotella nigrescens	Saliva	SR	ALDEX		1.59 (0.64; −1.57, 3.45)	Patient				TRUE
Prevotella nigrescens	Saliva	WGS	ANCOM	0.87 (± 0.17; 0.52, 1.21)		Patient			TRUE	TRUE
Prevotella nigrescens	Saliva	FL	ANCOM	1.74 (± 0.27; 1.19, 2.29)		Patient			TRUE	TRUE
Prevotella nigrescens	Saliva	SR	ANCOM	1.46 (± 0.35; 0.75, 2.17)		Patient			TRUE	TRUE
Prevotella nigrescens	Supragingival	FL	ANCOM	2.15 (± 0.16; 1.83, 2.46)		Patient	FL_sup	FALSE	TRUE	TRUE
Prevotella nigrescens	Supragingival	SR	ANCOM	1.39 (± 0.46; 0.45, 2.32)		Patient	SR_sup	TRUE	FALSE	FALSE
Streptococcus sp. oral taxon 431	Saliva	WGS	limma	−1.10 (± 0.33; −1.76, −0.44)		Control	WGS_sal	FALSE		FALSE
Streptococcus sp. oral taxon 431	Saliva	FL	limma	−1.25 (± 0.60; −2.46, −0.05)		Control	FL_sal	FALSE		FALSE
Streptococcus sp. oral taxon 431	Saliva	WGS	ANCOM	−0.43 (± 0.17; −0.77, −0.08)		Control			FALSE	FALSE
Streptococcus sp. oral taxon 431	Saliva	FL	ANCOM	−0.85 (± 0.28; −1.42, −0.28)		Control			FALSE	FALSE
Prevotella maculosa	Saliva	FL	limma	3.11 (± 0.98; 1.13, 5.09)		Patient	WGS_sal	FALSE		TRUE
Prevotella maculosa	Saliva	SR	limma	2.55 (± 0.50; 1.54, 3.55)		Patient	FL_sal	TRUE		TRUE
Prevotella maculosa	Saliva	FL	ALDEX		5.36 (0.99; −1.20, 2.77)	Patient	SR_sal	TRUE		TRUE
Prevotella maculosa	Saliva	SR	ALDEX		2.24 (0.87; −0.75, 4.75)	Patient				TRUE
Prevotella maculosa	Saliva	WGS	ANCOM	0.88 (± 0.10; 0.67, 1.09)		Patient			TRUE	TRUE
Prevotella maculosa	Saliva	FL	ANCOM	0.86 (± 0.11; 064, 1.07)		Patient			TRUE	TRUE
Prevotella maculosa	Saliva	SR	ANCOM	1.06 (± 0.32; 0.42, 1.70)		Patient			TRUE	TRUE
Streptococcus anginosus	Saliva	FL	limma	3.03 (± 0.86; 1.28, 4.78)		Patient	WGS_sal	FALSE		TRUE
Streptococcus anginosus	Saliva	SR	limma	2.20 (± 0.61; 0.97, 3.43)		Patient	FL_sal	TRUE		TRUE
Streptococcus anginosus	Saliva	FL	ALDEX		2.88 (0.72; −1.52, 3.54)	Patient	SR_sal	TRUE		TRUE
Streptococcus anginosus	Saliva	SR	ALDEX		1.78 (0.64; −0.59, 2.31)	Patient				TRUE
Streptococcus anginosus	Saliva	WGS	ANCOM	0.71 (± 0.12; 0.48, 0.95)		Patient			TRUE	FALSE
Streptococcus anginosus	Saliva	FL	ANCOM	0.61 (± 0.21; 0.18, 1.04)		Patient			TRUE	FALSE
Streptococcus anginosus	Saliva	SR	ANCOM	1.33 (± 0.28; 0.77, 1.89)		Patient			TRUE	TRUE
Streptococcus anginosus	Supragingival	FL	ANCOM	1.58 (± 0.22; 1.13, 2.02)		Patient	FL_sup	FALSE	TRUE	TRUE
Streptococcus anginosus	Supragingival	SR	ANCOM	1.39 (± 0.48; 0.41, 2.36)		Patient	SR_sup	FALSE	TRUE	FALSE
Anaeroglobus geminatus	Saliva	FL	limma	3.21 (± 0.84; 1.50, 4.91)		Patient	FL_sal	TRUE		TRUE
Anaeroglobus geminatus	Saliva	SR	limma	1.91 (± 0.70; 0.49, 3.32)		Patient	SR_sal	TRUE		TRUE
Anaeroglobus geminatus	Saliva	FL	ALDEX		5.33 (0.93; −1.63, 2.93)	Patient				TRUE
Anaeroglobus geminatus	Saliva	SR	ALDEX		1.47 (0.78; −1.87, 7.45)	Patient				TRUE
Anaeroglobus geminatus	Saliva	SR	ANCOM	0.87 (± 0.35; 0.16, 1.59)		Patient			TRUE	TRUE
Leptotrichia hofstadii	Saliva	FL	limma	2.83 (± 0.71; 1.40, 4.27)		Patient	WGS_sal	FALSE		TRUE
Leptotrichia hofstadii	Saliva	FL	ALDEX		4.37 (0.68; −1.48, 2.45)	Patient	FL_sal	TRUE		TRUE
Leptotrichia hofstadii	Saliva	WGS	ANCOM	0.58 (± 0.08; 0.42, 0.73)		Patient			TRUE	FALSE
Veillonella dispar	Saliva	FL	limma	−1.52 (± 0.59; −2.71, −0.32)		Control	WGS_sal	TRUE		TRUE
Veillonella dispar	Saliva	WGS	ANCOM	−0.84 (± 0.17; −1.18, −0.51)		Control	FL_sal	TRUE	FALSE	TRUE
Veillonella dispar	Saliva	FL	ANCOM	−1.04 (± 0.30; −1.64, −0.44)		Control	SR_sal	FALSE	TRUE	TRUE
Veillonella dispar	Saliva	SR	ANCOM	−0.88 (± 0.29; −1.45, −0.30)		Control			TRUE	FALSE
Selenomonas infelix	Saliva	FL	limma	2.27 (± 0.84; 0.57, 3.96)		Patient	FL_sal	TRUE		TRUE
Selenomonas infelix	Saliva	FL	ALDEX		3.26 (0.62; −2.01, 2.74)	Patient	SR_sal	TRUE		TRUE
Selenomonas infelix	Saliva	FL	ANCOM	0.73 (± 0.21; 0.30, 1.16)		Patient			TRUE	FALSE
Selenomonas infelix	Saliva	SR	ANCOM	0.60 (± 0.24; 0.12, 1.08)		Patient			TRUE	TRUE
Lachnoanaerobaculum orale	Saliva	FL	limma	−1.75 (± 0.69; −3.15, −0.35)		Control	FL_sal	FALSE		FALSE
Lachnoanaerobaculum orale	Saliva	FL	ANCOM	−0.89 (± 0.28; −1.46, −0.32)		Control	SR_sal	FALSE	TRUE	FALSE
Lachnoanaerobaculum orale	Saliva	SR	ANCOM	−0.98 (± 0.35; −1.69, −0.27)		Control			TRUE	FALSE
Streptococcus parasanguinis	Saliva	FL	limma	−1.42 (± 0.48; −2.40, −0.44)		Control	WGS_sal	TRUE		TRUE
Streptococcus parasanguinis	Saliva	SR	limma	−1.35 (± 0.34; −2.05, −0.66)		Control	FL_sal	FALSE		TRUE
Streptococcus parasanguinis	Saliva	WGS	ANCOM	−0.84 (± 0.19; −1.22, −0.45)		Control	SR_sal	FALSE	FALSE	TRUE
Streptococcus parasanguinis	Saliva	FL	ANCOM	−0.97 (± 0.32; −1.62, −0.31)		Control			FALSE	TRUE
Streptococcus parasanguinis	Saliva	SR	ANCOM	−1.08 (± 0.28; −1.66, −0.51)		Control			FALSE	FALSE
Streptococcus parasanguinis	Supragingival	FL	ANCOM	−0.65 (± 0.21; −1.07; −0.23)		Control	FL_sup	FALSE	TRUE	FALSE
Streptococcus sp. LPB0220	Saliva	FL	limma	−1.60 (± 0.59; −2.79, −0.42)		Control	WGS_sal	FALSE		TRUE
Streptococcus sp. LPB0220	Saliva	WGS	ANCOM	−0.78 (± 0.21; −1.20, −0.36)		Control	FL_sal	TRUE	FALSE	FALSE
Streptococcus sp. LPB0220	Saliva	FL	ANCOM	−0.94 (± 0.34; −1.64, −0.25)		Control	SR_sal	FALSE	TRUE	TRUE
Streptococcus sp. LPB0220	Saliva	SR	ANCOM	−0.96 (± 0.34; −1.66, −0.27)		Control			TRUE	FALSE
Selenomonas sp. oral taxon 920	Saliva	SR	limma	1.42 (± 0.35; 0.71, 2.13)		Patient	WGS_sal	FALSE		TRUE
Selenomonas sp. oral taxon 920	Saliva	FL	ALDEX		2.30 (0.85; −2.94, 4.96)	Patient	FL_sal	TRUE		TRUE
Selenomonas sp. oral taxon 920	Saliva	SR	ALDEX		1.69 (0.91; −0.97, 3.54)	Patient	SR_sal	TRUE		TRUE
Selenomonas sp. oral taxon 920	Saliva	WGS	ANCOM	1.02 (± 0.11; 0.80, 1.24)		Patient			TRUE	TRUE
Selenomonas sp. oral taxon 920	Saliva	FL	ANCOM	0.56 (± 0.22; 0.12, 1.00)		Patient			TRUE	FALSE
Selenomonas sp. oral taxon 920	Saliva	SR	ANCOM	0.86 (± 0.31; 0.23, 1.50)		Patient			TRUE	TRUE
Pseudopropionibacterium propionicum	Saliva	SR	limma	2.31 (± 0.73; 0.83, 3.80)		Patient	WGS_sal	FALSE		FALSE
Pseudopropionibacterium propionicum	Saliva	WGS	ANCOM	0.66 (± 0.16; 0.34, 0.99)		Patient	SR_sal	FALSE	TRUE	FALSE
Pseudopropionibacterium propionicum	Saliva	SR	ANCOM	0.86 (± 0.25; 0.37, 1.36)		Patient			FALSE	FALSE
Pseudopropionibacterium propionicum	Supragingival	FL	ANCOM	0.71 (± 0.09; 0.53, 0.90)		Patient	FL_sup	FALSE	TRUE	FALSE
Prevotella melaninogenica	Saliva	WGS	ANCOM	−0.46 (± 0.18; −0.82, −0.10)		Control	WGS_sal	FALSE	FALSE	FALSE
Prevotella melaninogenica	Saliva	FL	ANCOM	−0.71 (± 0.30; −1.32, −0.10)		Control	FL_sal	FALSE	TRUE	FALSE
Prevotella melaninogenica	Saliva	SR	ANCOM	−0.83 (± 0.22; −1.28, −0.37)		Control	SR_sal	FALSE	TRUE	FALSE
Leptotrichia buccalis	Saliva	WGS	ANCOM	0.67 (± 0.13; 0.42, 0.93)		Patient	WGS_sal	FALSE	TRUE	FALSE
Leptotrichia buccalis	Saliva	FL	ANCOM	0.66 (± 0.28; 0.11, 1.22)		Patient	FL_sal	FALSE	TRUE	FALSE
Leptotrichia buccalis	Supragingival	FL	ANCOM	0.71 (± 0.14; 0.43, 1.00)		Patient	FL_sup	FALSE	TRUE	FALSE
Mogibacterium timidum	Saliva	WGS	ANCOM	1.03 (± 0.08; 0.86, 1.20)		Patient	WGS_sal	FALSE	TRUE	FALSE
Mogibacterium timidum	Saliva	FL	ANCOM	1.01 (± 0.13; 0.74, 1.28)		Patient	FL_sal	FALSE	TRUE	FALSE
Mogibacterium timidum	Saliva	SR	ANCOM	0.71 (± 0.15; 0.40, 1.02)		Patient	SR_sal	FALSE	TRUE	TRUE
Mogibacterium timidum	Supragingival	FL	ANCOM	2.05 (± 0.11; 1.82, 2.28)		Patient	FL_sup	FALSE	TRUE	TRUE
Mogibacterium timidum	Supragingival	SR	ANCOM	0.57 (± 0.17; 0.23, 0.91)		Patient	SR_sup	FALSE	TRUE	FALSE
Olsenella uli	Saliva	WGS	ANCOM	6.97 (± 0.06; 6.85, 7.08)		Patient	WGS_sal	TRUE	TRUE	FALSE
Olsenella uli	Saliva	SR	ANCOM	1.29 (± 0.26; 0.77, 1.81)		Patient	SR_sal	FALSE	TRUE	FALSE
Olsenella uli	Supragingival	SR	ANCOM	0.97 (± 0.30; 0.36, 1.58)		Patient	SR_sup	TRUE	FALSE	FALSE
Dialister invisus	Saliva	WGS	ANCOM	0.54 (± 0.17; 0.20, 0.88)		Patient	WGS_sal	FALSE	TRUE	TRUE
Dialister invisus	Saliva	FL	ANCOM	0.71 (± 0.26; 0.18, 1.25)		Patient	FL_sal	FALSE	TRUE	FALSE
Dialister invisus	Supragingival	FL	ANCOM	1.10 (± 0.31; 0.49, 1.72)		Patient	FL_sup	TRUE	TRUE	FALSE
Dialister invisus	Supragingival	SR	ANCOM	0.97 (± 0.39; 0.18, 1.76)		Patient	SR_sup	FALSE	FALSE	FALSE
Schwartzia succinivorans	Saliva	FL	ANCOM	0.77 (± 0.15; 0.47, 1.07)		Patient	FL_sal	FALSE	TRUE	TRUE
Schwartzia succinivorans	Saliva	SR	ANCOM	0.86 (± 0.28; 0.30, 1.42)		Patient	SR_sal	TRUE	TRUE	TRUE
Schwartzia succinivorans	Supragingival	FL	ANCOM	0.87 (± 0.17; 0.53, 1.22)		Patient	FL_sup	TRUE	TRUE	TRUE
Schwartzia succinivorans	Supragingival	SR	ANCOM	1.45 (± 0.33; 0.79, 2.11)		Patient	SR_sup	TRUE	TRUE	TRUE
Streptococcus-anginosus-group-member	Saliva	FL	ANCOM	1.12 (± 0.20; 0.72, 1.53)		Patient	FL_sal	FALSE	TRUE	TRUE
Streptococcus-anginosus-group-member	Saliva	SR	ANCOM	0.68 (± 0.27; 0.14, 1.22)		Patient	SR_sal	FALSE	FALSE	TRUE
Streptococcus-anginosus-group-member	Supragingival	FL	ANCOM	0.89 (± 0.25; 0.38, 1.40)		Patient	FL_sup	FALSE	TRUE	FALSE

**Table 3:** Total intersection of all bacterial genera identified using ANCOM-BC2, Limma and ALDEx2 in salivary and supragingival samples from metagenomic, full-length and 16S short-read sequencing. The material, sequencing method, differential abundance analysis tool, log-fold-change with SE and 95% CI, respectively diff.btw with effect size and 95% CI for ALDEx2 and group with higher occurrence are shown. Also, the identification with LEfSe, the passing of the internal sensitivity analysis with pseudo-counts by ANCOM-BC2 and the passing of the cross-validation with inclusion of education years as additional covariate are shown. Abbreviations: ALDEx2, ANOVA-Like Differential Expression 2; ANCOM-BC2, Analysis of Compositions of Microbiomes with Bias Correction 2; CI, Confidence interval; LEfSe, Linear Discriminant Analysis Effect Size; Limma, Linear Models for Microarray; SE, Standard error

**Taxon**	**Material**	**Sequencing method**	**DA method**	**Log-fold-change (± SE; 95% CI)**	**diff.btw (effect; 95% CI)**	**Group**	**Identified with LEfSe**		**Passed sensitivity analysis**	**Identified with education years as covariate**
Anaeroglobus	Saliva	FL	limma	3.24 (± 0.99; 1.24, 5.25)		Patient	FL_sal	TRUE		TRUE
Anaeroglobus	Saliva	SR	limma	2.10 (± 0.59; 0.91, 3.29)		Patient	SR_sal	TRUE		TRUE
Anaeroglobus	Saliva	FL	ALDEX		4.87 (0.88; −1.70, 2.82)	Patient				TRUE
Anaeroglobus	Saliva	SR	ALDEX		1.46 (0.61; −1.38, 5.97)	Patient				TRUE
Anaeroglobus	Saliva	SR	ANCOM	0.88 (± 0.39; 0.18, 1.58)		Patient			TRUE	FALSE
Lactobacillus	Saliva	SR	limma	3.06 (± 0.64; 1.75, 4.36)		Patient	WGS_sal	FALSE		TRUE
Lactobacillus	Saliva	WGS	ANCOM	0.95 (± 0.18; 0.59, 1.32)		Patient	FL_sal	FALSE	TRUE	TRUE
Lactobacillus	Saliva	FL	ANCOM	1.81 (± 0.24; 1.32, 2.30)		Patient	SR_sal	FALSE	TRUE	TRUE
Lactobacillus	Saliva	SR	ANCOM	1.32 (± 0.50; 0.31, 2.32)		Patient			TRUE	FALSE
Lactobacillus	Supragingival	FL	ANCOM	2.24 (± 0.21; 1.81, 2.67)		Patient	FL_sup	FALSE	TRUE	TRUE
Olsenella	Saliva	SR	limma	2.05 (± 0.51; 1.02, 3.09)		Patient	WGS_sal	FALSE		TRUE
Olsenella	Saliva	SR	ALDEX		1.48 (0.61; −1.18, 3.16)	Patient	SR_sal	TRUE		TRUE
Olsenella	Saliva	WGS	ANCOM	0.96 (± 0.19; 0.58, 1.33)		Patient			TRUE	TRUE
Olsenella	Saliva	SR	ANCOM	1.22 (± 0.34; 0.53, 1.92)		Patient			TRUE	FALSE
Dialister	Saliva	SR	limma	1.32 (± 0.52; 0.27, 2.37)		Patient	WGS_sal	FALSE		FALSE
Dialister	Saliva	WGS	ANCOM	0.43 (± 0.15; 0.13, 0.73)		Patient	SR_sal	TRUE	TRUE	FALSE
Dialister	Saliva	SR	ANCOM	0.62 (± 0.27; 0.07, 1.16)		Patient			TRUE	FALSE
Dialister	Supragingival	FL	ANCOM	1.01 (± 0.31; 0.39, 1.64)		Patient	FL_sup	FALSE	FALSE	FALSE
Schwartzia	Saliva	SR	limma	2.20 (± 0.76; 0.66, 3.74)		Patient	FL_sal	FALSE		FALSE
Schwartzia	Saliva	FL	ANCOM	0.41 (± 0.16; 0.07, 0.74)		Patient	SR_sal	TRUE	TRUE	FALSE
Schwartzia	Saliva	SR	ANCOM	0.88 (± 0.27; 0.34, 1.41)		Patient			TRUE	FALSE
Schwartzia	Supragingival	FL	ANCOM	0.71 (± 0.17; 0.37, 1.05)		Patient	FL_sup	TRUE	TRUE	FALSE
Schwartzia	Supragingival	SR	ANCOM	1.15 (± 0.31; 0.52, 1.78)		Patient	SR_sup	TRUE	TRUE	FALSE
Streptococcus	Saliva	WGS	ANCOM	−0.52 (± 0.15; −0.82, −0.23)		Control	WGS_sal	FALSE	FALSE	FALSE
Streptococcus	Saliva	FL	ANCOM	−0.82 (± 0.23; −1.29, −0.36)		Control	FL_sal	FALSE	FALSE	FALSE
Streptococcus	Saliva	SR	ANCOM	−0.40 (± 0.15; −0.71, −0.09)		Control	SR_sal	FALSE	FALSE	FALSE
Veillonella	Saliva	WGS	ANCOM	−0.57 (± 0.15; −0.87, −0.27)		Control	WGS_sal	FALSE	FALSE	FALSE
Veillonella	Saliva	FL	ANCOM	−0.82 (± 0.20; −1.23, −0.42)		Control	FL_sal	FALSE	FALSE	FALSE
Veillonella	Saliva	SR	ANCOM	−0.46 (± 0.17; −0.81, −0.12)		Control	SR_sal	FALSE	FALSE	FALSE
Lachnoanaerobaculum	Saliva	WGS	ANCOM	−0.49 (± 0.15; −0.79, −0.19)		Control	WGS_sal	FALSE	TRUE	FALSE
Lachnoanaerobaculum	Saliva	FL	ANCOM	−1.02 (± 0.22; −1.47, −0.57)		Control	FL_sal	FALSE	TRUE	FALSE
Lachnoanaerobaculum	Saliva	SR	ANCOM	−0.65 (± 0.22; −1.09, −0.22)		Control	SR_sal	FALSE	TRUE	FALSE
Lachnoanaerobaculum	Supragingival	FL	ANCOM	−0.68 (± 0.23; −1.14, −0.22)		Control	FL_sup	FALSE	TRUE	FALSE
Lachnoanaerobaculum	Supragingival	SR	ANCOM	−0.94 (± 0.27; −1.49, −0.40)		Control	SR_sup	TRUE	TRUE	FALSE
Oribacterium	Saliva	WGS	ANCOM	−0.38 (± 0.14; −0.66, −0.11)		Control	WGS_sal	FALSE	TRUE	FALSE
Oribacterium	Saliva	FL	ANCOM	−0.89 (± 0.19; −1.27, −0.50)		Control	FL_sal	FALSE	TRUE	FALSE
Oribacterium	Saliva	SR	ANCOM	−0.59 (± 0.19; −0.99, −0.20)		Control	SR_sal	FALSE	TRUE	FALSE
Schaalia	Saliva	WGS	ANCOM	−0.86 (± 0.20; −1.26, −0.46)		Control	WGS_sal	FALSE	FALSE	FALSE
Schaalia	Saliva	FL	ANCOM	−0.83 (± 0.24; −1.31, −0.35)		Control	FL_sal	FALSE	TRUE	FALSE
Schaalia	Saliva	SR	ANCOM	−0.75 (± 0.22; −1.19, −0.31)		Control	SR_sal	FALSE	FALSE	FALSE
Schaalia	Supragingival	SR	ANCOM	−0.58 (± 0.21; −1.00, −0.16)		Control	SR_sup	FALSE	FALSE	FALSE
Campylobacter	Saliva	WGS	ANCOM	−0.35 (± 0.13; −0.62, −0.08)		Control	WGS_sal	FALSE	TRUE	FALSE
Campylobacter	Saliva	SR	ANCOM	−0.41 (± 0.16; −0.74, −0.09)		Control	SR_sal	FALSE	FALSE	FALSE
Campylobacter	Supragingival	SR	ANCOM	−0.53 (± 0.21; −0.96, −0.09)		Control	SR_sup	FALSE	TRUE	FALSE
Prevotella	Saliva	WGS	ANCOM	−0.42 (± 0.14; −0.71, −0.14)		Control	WGS_sal	FALSE	FALSE	FALSE
Prevotella	Saliva	FL	ANCOM	−0.75 (± 0.29; −1.34, −0.17)		Control	FL_sal	FALSE	TRUE	FALSE
Prevotella	Saliva	SR	ANCOM	−0.41 (± 0.13; −0.67, −0.15)		Control	SR_sal	FALSE	FALSE	FALSE

## Discussion

### General results and cross-validation

We identified differences between the oral microbiota of AD-patients and their cognitively healthy life partners. These differences were as far as we can tell, not primarily driven by oral health and were identified with three different sequencing approaches, with a certain intersection, but also variation. The most robust results were defined as 'Core dysbiosis'.

The Core results are further supported by two cross-validation approaches: A repetition of the main analysis with the addition of the education years as a covariate and an integrative analysis with the combined results and the sequencing method as additional covariate.

We realized that the education years as a covariate seemingly influenced the results of the metagenomic sequencing to a relatively large degree, but none of the other result sets. This observation was supported by GLMMMiRKAT, showing a significant association between the education years and the metagenomic data but none of the other datasets (see Supplementary Table 12).

The education years as a clinically meaningful parameter for AD can basically serve two functions: It can be seen as a risk factor representing what is called the cognitive reserve [74] or as an indicator for the socioeconomic background. As the role as a risk factor is redundant due to the pre-existing diagnosis and the close, in most cases long standing relationship between partners being probably a better indicator for socioeconomic situation we decided that incorporation of this covariate might result in an overadjustment detrimental to the overall results, especially as only one of five datasets was affected. However, as we did not want to ignore this effect at all, we used this expanded analysis to test the robustness of the Core dysbiosis. As mentioned before, most of the species 'passed' this test, although some were lost (see Tables 2, 3 and Supplementary Table 13–16). However, we still can not exclude the possibility that the difference in educational years reflects socioeconomic differences of patients and controls. Especially different sociodemographic backgrounds in early life phases could have influenced our results independently of the current socioeconomic status of the participants.

Our second cross-validation also confirmed the core results to a certain degree: Except for *Lachnoanaerbaculum orale, Leptotrichia buccalis, Olsenella uli, Dialister invisus, Schwartzia succinivorans* and *Streptococcus-anginosus-group-member* all species from the Core dysbiosis were reidentified when combining the input data although only *Streptococcus anginosus, Prevotella nigrescens, Prevotella maculosa, Leptotrichia hofstadii* and *Selenomonas infelix* could be identified with both methods (see Supplementary Table 11). This could be explained by the much stricter threshold for adjusted P values we chose for this cross-validation. However, as this approach was already exploratory at best, a conservative interpretation is recommended.

It should also be mentioned that we identified a significant difference in the BMI in our cohort with higher values in the control group. We did not find any significant association between the BMI and the microbial data by using GLMMMiRKAT (see Supplementary Table 12). Nevertheless, the weight and BMI should be briefly discussed as potential confounders influencing the differences observed in the microbiota.

It has been described that the oral microbiome could have a bidirectional connection to obesity and related metabolic disturbances via increased systemic inflammation while systemic diseases can on the other hand influence the immunological environment of the oral cavity [75]. Therefore, one could assume that a group with an increased BMI could represent with a more disease-associated microbiome. However, for older people and especially patients with dementia one also has to consider contrary connections as increasing age and low BMI increase the risk for sarcopenia [76] which has been associated with an increased risk for cognitive decline [77]. This reflects some of the general limitations of the BMI in certain populations [78].

It is therefore difficult to define the BMI as a clear protective or disease-promoting factor in our cohort. Considering that the mean BMI of the patients (23.73 kg/m^2^) was in the range of a normal BMI (18.5–25 kg/m^2^) and the mean BMI of the controls was only slightly above this cut-off (25.79 kg/m^2^) [78] (see Table 1) we think it is reasonable to say that the risk of the BMI largely influencing the differences in the microbial data is considerably low.

As mentioned in the introduction, there are biological [45–49], as well as statistical reasons [50] to prefer a comparison of paired samples in comparison to independent ones. In this study, the assumption of a more similar microbiota of people from the same household was confirmed by our analysis of the intra-partnership correlation and Bray–Curtis-distances. However, the differences between real partners and random pairings were more subtle than one could expect which is probably best explained by the fact that overall, the microbiota of a distinct anatomical niche still shares a lot of similarities between random people. Nevertheless, we were able to further strengthen the influence of the pairing on our final results by repeating the analysis with GLMMMiRKAT, Limma and ALDEx2 with a permutational approach by randomly changing the individual pairing while keeping the overall group comparison. For all three tests, P values increased after permutation although the effect was much more pronounced for GLMMMiRKAT than for Limma or ALDEx2 (see Supplementary Table 7,8,10). Nevertheless, this confirmation supports the assumption that microbial differences observed between partners have a higher probability of being associated with a disease state and not captured by chance, strengthening the main results of our study.

### Methodological advantages of a multi-level combined approach

Previous studies on the oral microbiota and AD showed high heterogeneity, with varying sequencing techniques, sampling methods, and statistics [2]. Most studies used 16S sequencing and the diagnosis of AD was made clinically, in contrast to the present study where different sequencing methods and the NIA-AA criteria were applied [2, 11]. To entangle the pathophysiology of AD further, it seems relevant to differentiate between dementia patients with biochemical confirmation of the AD diagnosis and without, since the exact pathogenesis of AD is multifactorial and complex [79, 80].

There is a consensus that microbiome studies come with a high degree of uncertainty: Long-read sequencing is known for higher error rates while short-read approaches have a questionable species-level resolution [38, 57]. The sample processing can be problematic from the collection method, over contamination, to different kits and taxonomic classifiers with individual databases [81]. Also, varying copy numbers of the 16S rRNA gene and amplification efficiency make 16S sequencing prone to amplification bias [41, 42, 81, 82]. Metagenomic sequencing of mucosal samples with over 90% of host DNA on the other hand is at risk of missing taxa [43, 44]. And lastly, methods for differential abundance analysis produce inconsistent results, not exclusively but among other things due to rarefication, filtering and varying statistical models and methods for multiple testing correction [83].

To overcome some of these problems, we decided to use a combinatorial approach with long- and short-read sequencing, 16S and metagenomic sequencing, as well as different differential abundance analysis methods and focus on recurrently identified taxa. Thereby, our results can be seen as more robust differences between the oral microbiota of AD-patients and healthy individuals. Also, the inclusion of life partners as controls reduces bias through cardiometabolic risk factors [48, 49] and allows the usage of paired testing which increases statistical power and potentially even compensates for the relatively small sample number [50].

By combining 16S short-read, 16S full-length and metagenomic sequencing, these methods theoretically complement each other: The amplification-based methods are prone to overrepresentation [41, 42, 81, 82] while the metagenomic sequencing could reflect the exact ratios yet is at risk of losing less abundant taxa [43, 44]. The 16S short-read sequencing has limited species-level resolution, which could be solved by using 16S full-length sequencing [57]. However, short-read sequencing is the more established technology, therefore differences in the results of 16S short-read and full-length sequencing observed in earlier studies [84] could be due to technical drawbacks of full-length sequencing.

By using LEfSe, as well as ANCOM-BC2, Limma and ALDEx2, we combined vastly different methods for differential abundance analysis [83]. LEfSe uses relative abundances and performs Kruskall-Wallis-rank-sum-tests for all taxa. Instead of following this up with a multiple testing correction, a linear discriminant analysis is performed, and the effect size of a taxon contributing to the overall group difference is quantified. Only taxa reaching a cut-off are defined as statistically significant, serving as an alternative approach to multiple testing correction [65]. However, the exclusive usage of this method has been criticized, as it tends to produce false-positive results [83].

The main reason for the inclusion of LEfSe in our analysis was to achieve a certain degree of comparability with other studies. While it is a widely utilized method for differential abundance analysis it lacks multiple testing correction and to the best of our knowledge does not offer any possibility for paired testing or incorporation of covariates, making it a poor tool to reflect our study design. However, taking into consideration the numerous problems when comparing microbiome studies, we decided to nevertheless include the results of this tool, as it is and has been widely used [81, 83], although we only incorporated it as an additional cross-validation. However, its results only overlapped to a small degree with the results of the other three methods supporting the recommendation to not use this method exclusively [83] (see Table 2).

ANCOM-BC2 on the other hand has been described as a conservative method with a low risk of false-positive results [83]. It uses non-normalized absolute read counts and prevalence filtering, log-transformation, and specific bias-correction to take into account differences in sequencing depth before performing multiple testing corrected hypothesis testing. It is potentially best served to work with varying sequencing depths and capable of modeling individual covariates influences. However, it lacks the capability of paired testing [66, 67].

In contrast, Limma, as well as ALDEx2, are both capable of paired testing and in case of Limma of incorporating covariates in the overall model [68–72]. ALDEx2 on the other hand has also been described as conservative with low risk of false-positive [83], probably reflected in the relatively low number of taxa identified with this tool. All three methods used in our Core analysis therefore complement each other in certain aspects, control the false-discovery rate individually and use approaches different enough to consider the overlap of their results as potentially more robust results.

One challenge when combining ANCOM-BC2, Limma and ALDEx2 is harmonizing low-abundance filtering criteria. Of these, only ANCOM-BC2 recommends a default prevalence cut-off of 0.1 [66, 67], which we adopted to satisfy its model assumptions. Neither Limma nor ALDEx2 prescribe such thresholds [68–72], so we determined their optimal cut-offs empirically via mean–variance plots. Consequently, Limma and ALDEx2 employed higher prevalence filters. We retained these differing thresholds because ANCOM-BC2 applies an additional structural-zero filtering step—excluding taxa absent in one group—which further eliminates low-abundance features. Since we omitted structural zeros from our main results, this secondary filter effectively compensates for the stricter prevalence criteria across methods, ensuring comparable Core dysbiosis profiles.

### Core results in relation to oral health and disease

This combinatorial approach seems to be confirmed by the bacteria identified as Core dysbiosis. Through literature review, we showed that the species and genera most consistently identified with a higher abundance in patients are associated with oral diseases as PD or caries. Several taxa identified in controls on the other hand play a role in oral health, inhibit pathogens, or are at least known commensals. Examples of literature supporting these associations are listed in Table 4.

**Table 4:** Species from the core dysbiosis, the group with their increased occurrence and selected literature references of their role in oral health and disease

Species from Core dysbiosis	Group presence	Role in oral health and disease
Prevotella nigrescens	Patients	- member of the PD-associated Orange-Complex [18, 19] - member of the PD-associated Prevotella-intermedia-group [85]
Prevotella maculosa	Patients	- has been described as saccharolytic and acid-producing [86] - higher abundances in the root canal of teeth with endodontic infections before compared to after therapy [87] - decreased in abundance after administration of a probiotic with known inhibitory effects on oral pathogens [88] - associated with gingivitis [89]
Streptococcus anginosus	Patients	- associated with PD [89] - higher abundance in supragingival plaque of children with caries compared to healthy controls [90] - isolated from teeth extracted due to caries [91] - one of the dominant taxa in infected root canals [92]
Streptococcus-anginosus-group-member	Patients	- member of the Streptococcus anginosus group consisting of Streptococcus anginosus/intermedius/constellatus [93] - Streptococcus constellatus has been described as PD-associated, while Streptococcus intermedius has been described as health-associated [89] - in other cases both have been described as periodontal pathogens [94] - Streptococcus intermedius is associated with persistent apical periodontitis [89]x
Anaeroglobus geminatus	Patients	- associated with PD [89] - induced an increased production of virulence factors when introduced in a polymicrobial biofilm in-vitro [95] - high abundance in root canals from infected teeth [96]
Leptotrichia buccalis	Patients	- associated with gingivitis [89] - isolated from tongue of subjects with oral malodour [97] - high abundance in infected root canals [98]
Leptotrichia hofstadii	Patients	- associated with gingivitis [89] - higher abundance of the genus Leptotrichia in children with caries compared to healthy controls [99] - higher abundance of the genus in patients with persistent, aggressive PD compared to successfully treated patients [100]
Selenomonas infelix	Patients	- associated with gingivitis [89] - can co-aggregate with Fusobacterium nucleatum [101] - showed immunostimulatory effect on Toll-like receptor comparable to in parallel identified periodontal pathogens [102]
Selenomonas sp. oral taxon 920	Patients	
Pseudopropionibacterium propionicum	Patients	- one of the most frequently identified species in root canals of teeth with apical periodontitis [103] - one of the dominant taxa in infected root canals [92] - has also been described as health-associated [89] - the genus is prevalent in persistent endodontic infections [104] - found in unsuccessfully treated endodontic lesions [105]
Mogibacterium timidum	Patients	- associated with PD [89] - isolated from periodontal abscess [106] - associated with persistent apical periodontitis [107] - found in higher abundance together with Porphyromonas gingivalis in subgingival plaque of patients with PD compared with healthy controls [108] - associated with clinical parameters for PD [109] - higher abundance in patients with refractory PD compared to healthy controls [110] - mentioned as one of the top species to define subgingival dysbiosis based on machine learning [111]
Olsenella uli	Patients	- one of the most frequently identified species in root canals of teeth with apical periodontitis [103] - associated with periodontal inflammation and tissue destruction [112] - one of the most frequently identified species in apical PD [113] - higher abundance in children with caries compared to healthy controls [114]
Dialister invisus	Patients	- associated with PD [89] - found in caries lesions [115] - mentioned as one of the most prevalent species in endodontic infections [116]
Schwartzia succinivorans	Patients	- several over Schwartzia-species associated with PD [89] - genus associated with PD [117]
Streptococcus sp. Oral taxon 431	Controls	
Veillonella dispar	Controls	- complex function for the genus in the oral microbiota [118], but often found in higher abundance in healthy individuals [119] and considered part of the healthy core microbiome [89]
Lachnoanaerobaculum orale	Controls	- lower abundance of the genus in subjects consuming more sugar-sweetened beverages [120, 121]
Streptococcus parasanguinis	Controls	- oral commensal inhibiting pathogens [122] - helps in forming a health-associated biofilm [123] - inhibits Streptococcus mutans in-vitro [124]
Streptococcus sp. LPB0220	Controls	- catalogued in the HOMD as Streptococcus parasanguinis HMT-721 [35]; however, it remains designated as Streptococcus sp. LPB0220 in GenBank (accession GCA_008727815.1) and related repositories
Prevotella melaninogenica	Controls	- described as an important member of the health-associated core microbiome [85] - has also been described as PD- and gingivitis-associated [89]

In general, highly prevalent genera as *Prevotella* and *Streptococcus* have species identified in patients and controls. However, the species of these genera belonging to the Core dysbiosis are either disease-associated and found in patients, or commensals and/or even health-associated and identified in controls. Both genera include many commensal species present in health and disease, but also some species with a distinct disease-association [85, 125]. Examples include the repeatedly identified and well described PD-pathogens *Prevotella nigrescens* [18, 19, 85], as well as *Streptococcus anginosus* and the associated members of the *Streptococcus-anginosus-group* [89–94]. In contrast, species described as part of a healthy Core microbiome as *Prevotella melaninogenica* [85] or taxa with direct inhibitory potential against pathogens as *Streptococcus parasanguinis* [122–124] are identified in controls.

Other disease-associated species in patients as *Dialister invisus, Anaeroglobus geminatus, Olsenella uli* and *Mogibacterium timidum* further support this trend [89, 95, 111–113]. Going beyond these Core taxa other species show similar distributions: *Prevotella loescheii* for example was repeatedly identified in higher abundance in patients, yet exclusively in the full-length data. This species is associated with periodontal abscess [126] and gingivitis [127]. It was also together with *Fusobacterium nucleatum* one of the most prevalent taxa in root canals and periapical teeth with post-treatment infections [128] and similarly together with *Prevotella nigrescens* one of the species from their genus with the highest capability for biofilm formation [127]. Other taxa which were repeatedly identified in patients yet did not fulfill the criteria for the Core dysbiosis are *Lancefieldella rimae* which has been described as a new periodontal pathogen [129] and *Bifidobacterium dentium* which is associated with PD [89] and often found in higher abundance in patients with caries [130–133].

Looking at the genus level, *Anaeroglobus, Olsenella* and *Dialister* are also found in higher levels in patients together with the highly caries-associated genus *Lactobacillus* [125, 134]. In contrast, the genus *Streptococcus* typically described as health-associated [122] shows a higher abundance in controls, further supported by lesser-known species as part of the Core dysbiosis as *Streptococcus sp. oral taxon 431* and *Streptococcus sp. LPB0220.* The latter one is catalogued in the Human Oral Microbiome Database (HOMD) as *Streptococcus parasanguinis HMT-721* [35] suggesting its potential role as a protective commensal [122–124]. However, it should be mentioned that these disease-associations are complex as for example the genus *Prevotella* shows a higher abundance in controls, while one of its most pathogenic members *Prevotella nigrescens* is clearly more prevalent in patients, probably showing the more opportunistic nature of many taxa [85].

Also, these observations so far do not include the central PD-pathogens *Treponema denticola*, *Tannerella forsythia* and PG of the disease-associated red complex [18, 19]. While PG did not show any difference at all, *Tannerella forsythia* and *Treponema denticola* were both identified in controls and patients across the different datasets showing no clear trend.

However, there are several practical explanations for the lack of detection of differences for these potential key factors of oral dysbiosis. First, the focus on saliva and supragingival swabs instead of subgingival samples might have underrepresented the abundance of PG as the pathophysiological process of PD is supposed to take place in the subgingival space. However, it is likely that besides this sampling issue the statistical approach is also likely to not have captured any differences for PG: Looking for example at the salivary data PG is present in the majority of samples, especially in the 16S short-read data with a prevalence of 100%. However, with its mean abundance never reaching even 0.1% a statistical approach like ours focusing on capturing the most robust differences is probably not ideally suited to identify more subtle differences. This observation underlines the general discrepancy in exploratory high-throughput analyses like ours which identify hundreds or thousands of features with the hope for narrowing them down to a more manageable number of relevant differences although this is often not in line with the statistical reality of these datasets. In future studies, the combination of high throughput analysis with more targeted approaches focusing on core features could help to solve this problem.

Other oral pathogens worth mentioning include *Fusobacterium nucleatum* [135] identified twice in patients, as well as *Filifactor alocis* and *Aggregatibacter actinomycetemcomitans* in controls. While *Filifactor alocis* has been described as a central PD-pathogen [136], *Aggregatibacter actinomycetemcomitans* was originally classified as part of the health-associated green complex [19]. However, it is now considered to be one of several members of the green complex that are also identified in PD [137]. This lack of a clear trend for the established pathogens matches our observation of a relatively normal oral status and no clear difference in oral health in our cohort and supports the hypothesis of an association of the oral microbiome and AD independent of at least PD diagnosis.

Although the comparability of microbiome related studies is limited, we nevertheless wanted to compare our results to other works in this field. Chaple-Gil et al., as well as Martínez-Martínez et al. just recently published their systematic reviews on the oral microbiota and dementia risk, respectively oral dysbiosis in AD. As mentioned before, it is challenging to identify a clear pattern of the oral microbiota in patients with AD. Nevertheless, both reviews generally agree that patients with AD/dementia present with higher abundances of bacteria of pathogenic nature. Although the exact results of the individual studies are far from consistent, a certain trend is recognizable and supported by the results of our work [12, 13].

For example, looking at the individual studies, the PD-associated species *Fusobacterium nucleatum* and its genus are regularly identified in patients, as it is the case in our study, although not as part of our Core dysbiosis [12, 138–140]. Species like *Anaeroglobus geminatus* and *Streptococcus anginosus* from our Core dysbiosis were previously found to be negatively correlated with cognitive function, as were the PD-associated *Dialister pneumosintes* [141] and *Campylobacter gracilis* [18], both also found to be elevated in patients in our data [142]. Similarly, the caries-associated genus *Lactobacillus* [125] together with its former members *Limosilactobacillus* and *Lacticaseibacillus* [143] were found in higher abundance in patients in our data as well as earlier studies [138]. On the other hand, species associated with oral health as *Haemophilus parainfluenza* [144] and *Streptococcus gordonii* [124] were found in controls in our cohort as well as other studies [12, 138, 142].

While these results are not completely consistent and even the disease-association of certain species is complex at best, for example from the genus *Prevotella* [89, 144], there is still a relatively large overlap especially in regard of the large heterogeneity of the studies involved. With the necessary caution some potential connections between the oral microbiome and central-nervous diseases could be seen: For example, with pathogenic *Prevotella* species regularly found in patients these could influence systemic inflammatory conditions and with these neurodegenerative diseases [145]. Swallowed with the saliva, this genus has also been associated with neurological disease as a part of the gastrointestinal microbiome [146].

Also interesting in this context is the fact that several of the species mentioned so far as AD-associated have been found in brain abscess, or subdural empyema: Species like *Dialister pneumosintes* [147], *Streptococcus intermedius* [148], or *Prevotella loescheii* [149, 150], which are not typically considered to be infectious agents in the central nervous system, therefore seemingly find their way from the oral cavity to the brain, at least indirectly strengthening the idea of a 'mouth-brain-axis'.

## Limitations

However, while we were able to identify a set of bacterial species with surprisingly clear pathological nature in the oral microbiota of AD-patients the cross-sectional design of our study still forbids any causal conclusions. Also, one limitation of this study is the low ethnic diversity of the sample as participants were recruited in Düsseldorf only. This could limit the generalizability of the results. Future studies should ensure greater demographic and ethnic diversity to examine potential differences in outcomes between population groups.

Also important to mention is the fact that we did not control for the diet or medication of the participants. Although it is well described that the diet and the medication influence the oral microbiome [151, 152] it is extremely difficult to effectively control these factors, especially in the context of AD: While a controlled feeding study was beyond the limits of what was possible in this study even food questionnaires could have only produce questionable results due to the cognitive state of the patient group. However, it should be mentioned that the inclusion of life partners has the potential to at least control for individual lifestyle and health decisions to a certain degree [48, 49] although the influence of a healthy/unhealthy lifestyle over the lifespan remains a potential confounder.

The individual medication regimen on the other hand might be an easier factor to control. However, due to the mean age of our cohort nearly all participants regularly took certain medications introducing potential confounders. Especially the intake of certain drugs might influence the results with Hisamatsu et al. for example just recently showing that especially drugs from the class of acetycholinesterase-inhibitors influence the salivary microbiome [153]. However, excluding patients taking these drugs would have been problematic from an ethical point of view and beyond the scope of our work as acetylcholinesterase-inhibitors are recommended as first-line drugs in Germany for the treatment of AD and therefore taken by the majority of the patients [154]. Incorporating the intake of these drugs as an additional covariate on the other hand would have probably led to overadjustment with the drug intake effectively serving as a proxy for the grouping [155].

Although the general comparability of the results from Hisamatsu et al. and our study is limited among other things by the use of different statistical approaches, we nevertheless wanted to assess how much of our results could be explained by the observed medication effect. When comparing our findings to those of Hisamatsu et al., some parallels can be seen. However, there is no consistent trend across both datasets: The genus *Streptococcus* was less abundant with the intake of acetylcholinesterase-inhibitors or anti-dementia drugs in general, which would be in line with our observation of higher abundance of this taxon in controls (see Table 3). Similarly, the authors found higher levels of *Lactobacillus* and lower levels of *Haemophilus* and *Granulicatella* in individuals taking NMDA-receptor-antagonists (N-methyl-D-aspartate). While this would also be roughly consistent with our results (see Table 3 and Supplementary Table 9), it should be noted that only three of our patients were taking this drug class. In contrast, the vast majority received other drugs from the class of acetylcholinesterase inhibitors which, although showing a similar trend, were not significantly associated with the aforementioned taxa in the analysis by Hisamatsu et al. Furthermore, the most consistently identified taxon, *G_undefined_Clostridiales Family XIII Incertae Sedis*, was absent from our results, with only the order *Clostridiales* identified – but showing a trend opposite to the results of Hisamatsu et al. (see Supplementary Table 9). However, the authors also identified several taxa exclusively associated with cognitive state. Among these, the higher abundance of *Treponema* in patients aligned with our observations, as did the higher abundances of *Fusobacterium* and *Capnocytophaga* when comparing patients with dementia and mild cognitive impairment (see Supplementary Table 9). In contrast, Hisamatsu et al. also reported a higher abundance of the genus *Alloprevotella* in dementia patients, which we did not detect at all. Instead, we found the closely related genus *Prevotella* to be higher in controls, although several of its members were simultaneously more abundant in patients (see Tables 2 and 3) [153].

Nevertheless, it cannot be excluded that the differences observed between patients and controls in our study are potentially driven by medication rather than disease status. In particular, the difference in the abundance of *Streptococcus* and to a lesser extent *Lactobacillus* could plausibly be explained by medication effects, based on the observations of Hisamatsu et al. Larger cohorts including substantial numbers of patients with and without medication are therefore necessary to accurately model these potential influences in future studies.

Another aspect which can be seen as a limitation of this study is the choice of the sampling material. Although we already included two different sampling materials, neither saliva, nor supragingival swabs are probably ideally suited to reflect the local inflammatory process in the subgingival sulcus [156]. Therefore, incorporating this sampling material would have been a valuable addition to further strengthening a possible connection of PD, AD and the oral microbiome. However, we nevertheless decided to avoid this sampling method in the context of this exploratory work, mainly for two reasons: First, of the different sampling methods used in oral microbiome studies subgingival sampling tends to be the most biased by reproducibility problems. Even in the limited context of investigating the oral microbiome in neurodegenerative diseases studies used paper tips and curettes [140, 157], sometimes pooled their samples [157] and collected the material either from the most representative periodontal pocket, or different index locations [142, 157]. Secondly, while prior work investigating the association of PD and AD laid the groundwork for studies like ours we were not primarily interested in the connection of these two diagnoses but rather a potential association of a shifting oral ecosystem and AD. Saliva offers good reproducibility, potentially reflects pathological processes of the oral cavity as a whole [37] and showed a consistently high DNA-yield comfortably allowing sequencing exactly the same sample with different methods thereby further reducing batch effects potentially occuring through repetitive measuring.

Another complex topic is the interpretation of species-level results, especially for the 16S sequencing approaches. There is a consensus that 16S sequencing has only a questionable species-level resolution which is one reason why metagenomic sequencing could be the superior technology [40]. Nevertheless, as mentioned before, metagenomically sequenced mucosal samples can lack the necessary sequencing depth of bacterial reads [43, 44]. For this reason, it is often recommended to focus on the genus-level instead [40].

However, we hypothesized that by using a combined methodological approach and focusing on the most robust features in our dataset, we can overcome these concerns at least to a certain degree. This assumption is supported by the correlation between the sequencing methods analyzed via Mantel-test which, regardless of the specific correlation method used, produced the highest correlations for the species-level, followed by the genus-level (see Supplementary Table 17). However, one general concern at least for pairwise correlation of microbiome datasets is that they are not well suited for especially Pearson-correlation due to their sparse nature and lack of normality. Although this can be overcome to a certain degree by usage of the rank-based Spearman-correlation and the Bray–Curtis-based Mantel-test [73], we nevertheless repeated the tests after filtering by prevalence, respectively mean abundance. As expected, the correlation results dropped most for the species-level as these data had the most low-abundance/-prevalence entries. However, even after filtering, the correlation between sequencing methods remained high for both the species- and genus-level. Similarly, looking at the overlap in taxon-identification we can see that regardless of the filtering the percentage of overlap is nearly identical for both the species- and genus-level and includes the vast majority of taxa after filtering by minimum mean abundance (see Figs. 10 and 11). These results support our assumption that at least for our dataset the central features are identified by all sequencing methods, making the species-level results more reliable. Although still not a perfect approach, the Core results of our study support this overall assumption: Most of the repeatedly identified species were present in all three sequencing methods for the saliva, respectively two for the supragingival samples. However, some taxa still show some clear between methods differences as for example the species *Lachnoanaerobaculum orale* and *Selenomonas infelix* or the genus *Schwartzia* are missing in the metagenomic data (see Figs. 12 and 13). This might indeed be the consequence of misclassification, for example in the case of *Lachnoanaerobaculum orale*. Looking at the genus-level *Lachnoanaerobaculum* is present in all three datasets with a consistently higher abundance in controls across all three sequencing methods. At the species-level however, *L.orale* is missing completely in the metagenomic data, although other species like *L.umeanse* and *saburreum* were identified, with the latter showing the significant difference 'missing' for *L.orale*. Taking into consideration the close genetic relations between these species [158] it seems reasonable to consider a misclassification having taken place here.

Nevertheless, the majority of the Core dysbiosis taxa were identifed with all three sequencing methods, followed by vigorous statistical testing which is why we assume that with the necessary caution even the species-level results can be interpreted with a high degree of certainty.

In addition to the aforementioned limitations, future studies could try to use more targeted approaches to investigate the species identified in this study and/or use a longitudinal design, more rigorous inclusion criteria and more detailed dental diagnostics to identify potential causal relations of the oral microbiome and AD.

## Conclusions

To the best of our knowledge, this is the first study investigating the oral microbiota of AD-patients and healthy controls in a paired setting and with a combinatorial approach. With the necessary caution, we can conclude that our results further strengthen the observation of a dysbiotic oral community in AD-patients. We further conclude that a combined usage of different methods and focus on the intersection might be a feasible way to overcome some of the individual drawbacks of the methods included.

## Supplementary Information

Supplementary Material 1: Metadata files 

Supplementary Material 2: Supplementary scripts

Supplementary Material 3: Supplementary Table 1

Supplementary Material 4: Supplementary Table 2

Supplementary Material 5: Supplementary Table 3

Supplementary Material 6: Supplementary Table 4

Supplementary Material 7: Supplementary Table 5

Supplementary Material 8: Supplementary Table 6

Supplementary Material 9: Supplementary Table 7

Supplementary Material 10: Supplementary Table 8

Supplementary Material 11: Supplementary Table 9

Supplementary Material 12: Supplementary Table 10

Supplementary Material 13: Supplementary Table 11

Supplementary Material 14: Supplementary Table 12

Supplementary Material 15: Supplementary Table 13

Supplementary Material 16: Supplementary Table 14

Supplementary Material 17: Supplementary Table 15

Supplementary Material 18: Supplementary Table 16

Supplementary Material 19: Supplementary Table 17

Supplementary Material 20: Supplementary Table 18

## Data Availability

The datasets supporting the conclusions of this article are available in the NCBI Sequence Read Archive (SRA) database under BioProject accession number PRJNA1243403 (https://www.ncbi.nlm.nih.gov/bioproject/PRJNA1243403).

## Abbreviations

AD: Alzheimer's disease
ALDEx2: ANOVA-Like Differential Expression 2
ANCOM-BC2: Analysis of Compositions of Microbiomes with Bias Correction 2
ANOVA: Analysis of Variance
BMFZ: Biological and Medical Research Center
BMI: Body-Mass-Index
bp: Base pair
Bracken: Bayesian Reestimation of Abundance with KrakEN
C: Control
CI: Confidence interval
CSF: Cerebro-spinal-fluid
DA: Differential abundance
DNA: Deoxyribonucleic acid
dsDNA: Double strain DNA
gDNA: Genomic DNA
Educ: Education
FDI: Fédération Dentaire Internationale
FDR: False discovery rate
FL: Full-length
GLMMMiRKAT: Generalized Linear Mixed Model Microbiome Regression-based Kernel Association Test
GTL: Genomics and Trandscriptomics Laboratory
HOMD: Human Oral Microbiome Database
LDA: Linear Discriminant Analysis
LEfSe: Linear Discriminant Analysis Effect Size
lfc: Log-fold-change
Limma: Linear Models for Microarray
LPS: Lipopolysaccharide
lvha: Low variance, high abundance
LVR: Landesverband Rheinland
MiRKAT: Microbiome Regression-based Kernel Association Test
MMSE: Mini Mental Status Examination
NCBI: National Center for Biotechnology Information
NGS: Next-generation sequencing
NIA-AA: National Institute of Aging and Alzheimer’s Association
NMDA: N-methyl-D-aspartate
P: Patient
PCoA: Principal Coordinate Analysis
PCR: Polymerase chain reaction
PD: Periodontal disease
PERMANOVA: Permutational Multivariate Analysis Of Variance
PG: Porphyromonas gingivalis
Pseudo: Pseudopropionibacterium
RNA: Ribonucleic acid
rrnDB: Ribosomal RNA Operon Copy Number Database
rRNA: Ribosomal RNA
SBI: Sulcus bleeding index
SE: Standard error
Sel: Selenomonas
sp: Species
SR: Short-Read
SRA: Sequence Read Archive
SRS: Scaling with Ranked Subsampling
Strp: Streptococcus
Supra: Supragingival
TMMwsp: Trimmed Mean of M-values with Singleton Pairing
V3/V4: Variable region 3 /4
WGS: Whole-genome-sequencing

## References

[1] Nichols E, Steinmetz JD, Vollset SE, Fukutaki K, Chalek J, Abd-Allah F, et al. Estimation of the global prevalence of dementia in 2019 and forecasted prevalence in 2050: an analysis for the Global Burden of Disease Study 2019. The Lancet Public Health. 2022;7(2):e105–25. 34998485 10.1016/S2468-2667(21)00249-8 PMC8810394

[2] Weber C, Dilthey A, Finzer P. The role of microbiome-host interactions in the development of Alzheimer´s disease. Front Cell Infect Microbiol. 2023;13:1151021. 37333848 10.3389/fcimb.2023.1151021 PMC10272569

[3] Andrews SJ, Renton AE, Fulton-Howard B, Podlesny-Drabiniok A, Marcora E, Goate AM. The complex genetic architecture of Alzheimer’s disease: novel insights and future directions. EBioMedicine. 2023;90:104511. 36907103 10.1016/j.ebiom.2023.104511 PMC10024184

[4] Eiser AR, Fulop T. Alzheimer’s disease is a multi-organ disorder: it may already be preventable. J Alzheimers Dis. 2023;91(4):1277–81. 36617785 10.3233/JAD-221078

[5] Chen CK, Wu YT, Chang YC. Association between chronic periodontitis and the risk of Alzheimer’s disease: a retrospective, population-based, matched-cohort study. Alzheimers Res Ther. 2017;9:56. 28784164 10.1186/s13195-017-0282-6 PMC5547465

[6] Kamer AR, Pirraglia E, Tsui W, Rusinek H, Vallabhajosula S, Mosconi L, et al. Periodontal disease associates with higher brain amyloid load in normal elderly. Neurobiol Aging. 2015;36:627–33. 25491073 10.1016/j.neurobiolaging.2014.10.038 PMC4399973

[7] Sung CE, Huang RY, Cheng WC, Kao TW, Chen WL. Association between periodontitis and cognitive impairment: analysis of national health and nutrition examination survey (NHANES) III. J Clin Periodontol. 2019;46:790–8. 31152592 10.1111/jcpe.13155

[8] Petersen C, Round JL. Defining dysbiosis and its influence on host immunity and disease. Cell Microbiol. 2014;16(7):1024–33. 24798552 10.1111/cmi.12308 PMC4143175

[9] Abusleme L, Hoare A, Hong BY, Diaz PI. Microbial signatures of health, gingivitis, and periodontitis. Periodontol 2000. 2021;86(1):57–78. 33690899 10.1111/prd.12362

[10] Singhrao SK, Harding A. Is Alzheimer’s disease a polymicrobial host microbiome dysbiosis? Expert Rev Anti-Infect Ther. 2020;18(4):275–7. 32048530 10.1080/14787210.2020.1729741

[11] Liu S, Dashper SG, Zhao R. Association between oral bacteria and Alzheimer’s disease: a systematic review and meta-analysis. J Alzheimers Dis. 2023;91(1):129–50. 36404545 10.3233/JAD-220627

[12] Chaple-Gil AM, Santiesteban-Velázquez M, Urbizo Vélez JJ. Association between oral microbiota dysbiosis and the risk of dementia: a systematic review. Dentistry journal. 2025;13(6):227. 40559130 10.3390/dj13060227 PMC12191743

[13] Martínez-Martínez V, Rodríguez-Lozano FJ, Pecci-Lloret MP, Pérez-Guzmán N. Association between oral dysbiosis and Alzheimer’s disease: a systematic review. J Clin Med. 2025;14(10):3415. 40429409 10.3390/jcm14103415 PMC12111987

[14] Almagbol M. Periodontitis and systemic inflammatory multimorbidity: narrative review of literature. J Pharm Bioallied Sci. 2025;17(Suppl 3):S2067–70. 41164655 10.4103/jpbs.jpbs_1855_24 PMC12563893

[15] Li X, Kiprowska M, Kansara T, Kansara P, Li P. Neuroinflammation: a distal consequence of periodontitis. J Dent Res. 2022;101(12):1441–9. 35708472 10.1177/00220345221102084 PMC9608094

[16] Galea I. The blood-brain barrier in systemic infection and inflammation. Cell Mol Immunol. 2021;18(11):2489–501. 34594000 10.1038/s41423-021-00757-x PMC8481764

[17] Țica O, Romanul I, Ciavoi G, Pantea VA, Scrobota I, Șipoș L, et al. A clinical review of the connections between diabetes mellitus, periodontal disease, and cardiovascular pathologies. Biomedicines. 2025;13(9):2309. 41007869 10.3390/biomedicines13092309 PMC12467886

[18] Abdulkareem AA, Al-Taweel FB, Al-Sharqi AJB, Gul SS, Sha A, Chapple ILC. Current concepts in the pathogenesis of periodontitis: from symbiosis to dysbiosis. J Oral Microbiol. 2023;15(1):2197779. 37025387 10.1080/20002297.2023.2197779 PMC10071981

[19] Socransky SS, Haffajee AD, Cugini MA, Smith C, Kent RLJ. Microbial complexes in subgingival plaque. J Clin Periodontol. 1998;25(2):134–44. 9495612 10.1111/j.1600-051x.1998.tb02419.x

[20] Dominy SS, Lynch C, Ermini F, Benedyk M, Marczyk A, Konradi A, et al. *Porphyromonas gingivalis* in Alzheimer’s disease brains: evidence for disease causation and treatment with small-molecule inhibitors. Sci Adv. 2019;5(1):eaau3333. 30746447 10.1126/sciadv.aau3333 PMC6357742

[21] Poole S, Singhrao SK, Kesavalu L, Curtis MA, Crean S. Determining the presence of periodontopathic virulence factors in short-term postmortem Alzheimer’s disease brain tissue. J Alzheimers Dis. 2013;36(4):665–77. 23666172 10.3233/JAD-121918

[22] Ding Y, Ren J, Yu H, Yu W, Zhou Y. *Porphyromonas gingivalis*, a periodontitis causing bacterium, induces memory impairment and age-dependent neuroinflammation in mice. Immunity Ageing. 2018;15:6. 29422938 10.1186/s12979-017-0110-7 PMC5791180

[23] Ilievski V, Zuchowska PK, Green SJ, Toth PT, Ragozzino ME, Le K, et al. Chronic oral application of a periodontal pathogen results in brain inflammation, neurodegeneration and amyloid beta production in wild type mice. PLoS ONE. 2018;13(10):e0204941. 30281647 10.1371/journal.pone.0204941 PMC6169940

[24] Kaba S, Lynch C, Raha D, et al. Clinical Trials and Aging: 11th Conference Clinical Trials on Alzheimer’s Disease, October 24–27, 2018, Barcelona, Spain. The Journal Of Prevention of Alzheimer’s Disease. 2018;5:1–151.

[25] Sabbagh MN, Decourt B. COR388 (atuzaginstat): an investigational gingipain inhibitor for the treatment of Alzheimer disease. Expert Opin Investig Drugs. 2022;31(10):987–93. 36003033 10.1080/13543784.2022.2117605 PMC10275298

[26] Hajishengallis G, Darveau RP, Curtis MA. The keystone-pathogen hypothesis. Nat Rev Microbiol. 2012;10(10):717–25. 22941505 10.1038/nrmicro2873 PMC3498498

[27] Darveau RP. Periodontitis: a polymicrobial disruption of host homeostasis. Nat Rev Microbiol. 2010;8(7):481–90. 20514045 10.1038/nrmicro2337

[28] Olczak T, Simpson W, Liu X, Genco CA. Iron and heme utilization in *Porphyromonas gingivalis*. FEMS Microbiol Rev. 2005;29(1):119–44. 15652979 10.1016/j.femsre.2004.09.001

[29] Hajishengallis G, Liang S, Payne MA, Hashim A, Jotwani R, Eskan MA, et al. Low-abundance biofilm species orchestrates inflammatory periodontal disease through the commensal microbiota and complement. Cell Host Microbe. 2011;10(5):497–506. 22036469 10.1016/j.chom.2011.10.006 PMC3221781

[30] Nara PL, Sindelar D, Penn MS, Potempa J, Griffin WST. *Porphyromonas gingivalis* outer membrane vesicles as the major driver of and explanation for neuropathogenesis, the cholinergic hypothesis, iron dyshomeostasis, and salivary lactoferrin in Alzheimer’s disease. J Alzheimers Dis. 2021;82(4):1417–50. 34275903 10.3233/JAD-210448 PMC8461682

[31] Singhrao SK, Olsen I. Are *Porphyromonas gingivalis* outer membrane vesicles microbullets for sporadic Alzheimer’s disease manifestation? J Alzheimers Dis Rep. 2018;2(1):219–28. 30599043 10.3233/ADR-180080 PMC6311351

[32] Elashiry M, Carroll A, Yuan J, Liu Y, Hamrick M, Cutler CW, et al. Oral microbially-induced small extracellular vesicles cross the blood-brain barrier. Int J Mol Sci. 2024;25(8):4509. 10.3390/ijms25084509 PMC11049816 38674094

[33] Gong T, Chen Q, Mao H, Zhang Y, Ren H, Xu M, et al. Outer membrane vesicles of *Porphyromonas gingivalis* trigger NLRP3 inflammasome and induce neuroinflammation, tau phosphorylation, and memory dysfunction in mice. Front Cell Infect Microbiol. 2022;12:925435. 36017373 10.3389/fcimb.2022.925435 PMC9397999

[34] Lauritano D, Moreo G, Della Vella F, Di Stasio D, Carinci F, Lucchese A, et al. Oral Health status and need for oral care in an aging population: a systematic review. Int J Environ Res Public Health. 2019;16(22):4558. 31752149 10.3390/ijerph16224558 PMC6888624

[35] Escapa IF, Chen T, Huang Y, Gajare P, Dewhirst FE, Lemon KP. New insights into human nostril microbiome from the expanded human oral microbiome database (eHOMD): a resource for the microbiome of the human aerodigestive tract. Msystems. 2018;3(6):e00187-e218. 30534599 10.1128/mSystems.00187-18 PMC6280432

[36] Segata N, Haake SK, Mannon P, Lemon KP, Waldron L, Gevers D, et al. Composition of the adult digestive tract bacterial microbiome based on seven mouth surfaces, tonsils, throat and stool samples. Genome Biol. 2012;13(6):R42. 22698087 10.1186/gb-2012-13-6-r42 PMC3446314

[37] Belstrøm D. The salivary microbiota in health and disease. J Oral Microbiol. 2020;12(1):1723975. 32128039 10.1080/20002297.2020.1723975 PMC7034443

[38] Hu T, Chitnis N, Monos D, Dinh A. Next-generation sequencing technologies: an overview. Hum Immunol. 2021;82(11):801–11. 33745759 10.1016/j.humimm.2021.02.012

[39] Regueira-Iglesias A, Balsa-Castro C, Blanco-Pintos T, Tomás I. Critical review of 16S rRNA gene sequencing workflow in microbiome studies: from primer selection to advanced data analysis. Mol Oral Microbiol. 2023;38(5):347–99. 37804481 10.1111/omi.12434

[40] Wensel CR, Pluznick JL, Salzberg SL, Sears CL. Next-generation sequencing: insights to advance clinical investigations of the microbiome. J Clin Invest. 2022;132(7):e154944. 35362479 10.1172/JCI154944 PMC8970668

[41] Bars-Cortina D, Ramon E, Rius-Sansalvador B, Guinó E, Garcia-Serrano A, Mach N, et al. Comparison between 16S rRNA and shotgun sequencing in colorectal cancer, advanced colorectal lesions, and healthy human gut microbiota. BMC Genomics. 2024;25(1):730. 39075388 10.1186/s12864-024-10621-7 PMC11285316

[42] Durazzi F, Sala C, Castellani G, Manfreda G, Remondini D, De Cesare A. Comparison between 16S rRNA and shotgun sequencing data for the taxonomic characterization of the gut microbiota. Sci Rep. 2021;11(1):3030. 33542369 10.1038/s41598-021-82726-y PMC7862389

[43] Pereira-Marques J, Hout A, Ferreira RM, Weber M, Pinto-Ribeiro I, van Doorn LJ, et al. Impact of host DNA and sequencing depth on the taxonomic resolution of whole metagenome sequencing for microbiome analysis. Front Microbiol. 2019;10:1277. 31244801 10.3389/fmicb.2019.01277 PMC6581681

[44] Quince C, Walker AW, Simpson JT, Loman NJ, Segata N. Shotgun metagenomics, from sampling to analysis. Nat Biotechnol. 2017;35(9):833–44. 28898207 10.1038/nbt.3935

[45] Cheng Q, Krajmalnik-Brown R, DiBaise JK, Maldonado J, Guest MA, Todd M, et al. Relationship functioning and gut microbiota composition among older adult couples. Int J Environ Res Public Health. 2023;20(8):5435. 37107717 10.3390/ijerph20085435 PMC10138905

[46] Wang H, Yang M, Cheng S, Ren Y, Deng Y, Liang J, et al. The spouses of stroke patients have a similar oral microbiome to their partners with an elevated risk of stroke. Microorganisms. 2022;10(11):2288. 36422358 10.3390/microorganisms10112288 PMC9697374

[47] Dill-McFarland KA, Tang ZZ, Kemis JH, Kerby RL, Chen G, Palloni A, et al. Close social relationships correlate with human gut microbiota composition. Sci Rep. 2019;9(1):703. 30679677 10.1038/s41598-018-37298-9 PMC6345772

[48] Lin B, Pan L, He H, Hu Y, Tu J, Zhang L, et al. Spousal similarities in cardiovascular risk factors in Northern China: a community-based cross-sectional study. Int J Public Health. 2023;68:1605620. 36895713 10.3389/ijph.2023.1605620 PMC9988901

[49] Nakaya N, Xie T, Scheerder B, Tsuchiya N, Narita A, Nakamura T, et al. Spousal similarities in cardiometabolic risk factors: a cross-sectional comparison between Dutch and Japanese data from two large biobank studies. Atherosclerosis. 2021;334:85–92. 34492521 10.1016/j.atherosclerosis.2021.08.037

[50] Stevens JR, Herrick JS, Wolff RK, Slattery ML. Power in pairs: assessing the statistical value of paired samples in tests for differential expression. BMC Genomics. 2018;19(1):953. 30572829 10.1186/s12864-018-5236-2 PMC6302489

[51] Albert MS, DeKosky ST, Dickson D, Dubois B, Feldman HH, Fox NC, et al. The diagnosis of mild cognitive impairment due to Alzheimer’s disease: recommendations from the National Institute on Aging-Alzheimer’s Association workgroups on diagnostic guidelines for Alzheimer’s disease. Alzheimers Dement. 2011;7(3):270–9. 21514249 10.1016/j.jalz.2011.03.008 PMC3312027

[52] Dubois B, Feldman HH, Jacova C, Hampel H, Molinuevo JL, Blennow K, et al. Advancing research diagnostic criteria for Alzheimer’s disease: the IWG-2 criteria. Lancet Neurol. 2014;13(6):614–29. 24849862 10.1016/S1474-4422(14)70090-0

[53] Folstein MF, Folstein SE, McHugh PR. “Mini-mental state”. A practical method for grading the cognitive state of patients for the clinician. J Psychiatr Res. 1975;12(3):189–98. 1202204 10.1016/0022-3956(75)90026-6

[54] Suzuki S, Sugihara N, Kamijo H, Morita M, Kawato T, Tsuneishi M, et al. Reasons for tooth extractions in Japan: the second nationwide survey. Int Dent J. 2022;72(3):366–72. 34193342 10.1016/j.identj.2021.05.008 PMC9275201

[55] Turp JC, Alt KW. Designating teeth: the advantages of the FDI’s two-digit system. Quintessence international (Berlin, Germany : 1985). 1995;26(7):501–4. 8935036

[56] Lange DE, Plagmann HC, Eenboom A, Promesberger A. [Clinical methods for the objective evaluation of oral hygiene]. Dtsch Zahnarztl Z. 1977;32(1):44–7. 264444

[57] Curry KD, Wang Q, Nute MG, Tyshaieva A, Reeves E, Soriano S, et al. Emu: species-level microbial community profiling of full-length 16S rRNA Oxford Nanopore sequencing data. Nat Methods. 2022;19(7):845–53. 35773532 10.1038/s41592-022-01520-4 PMC9939874

[58] Stoddard SF, Smith BJ, Hein R, Roller BRK, Schmidt TM. rrnDB: improved tools for interpreting rRNA gene abundance in bacteria and archaea and a new foundation for future development. Nucleic Acids Res. 2015;43(Database issue):D593–8. 25414355 10.1093/nar/gku1201 PMC4383981

[59] O’Leary NA, Wright MW, Brister JR, Ciufo S, Haddad D, McVeigh R, et al. Reference sequence (RefSeq) database at NCBI: current status, taxonomic expansion, and functional annotation. Nucleic Acids Res. 2016;44(D1):D733–45. 26553804 10.1093/nar/gkv1189 PMC4702849

[60] Wood DE, Lu J, Langmead B. Improved metagenomic analysis with Kraken 2. Genome Biol. 2019;20(1):257. 31779668 10.1186/s13059-019-1891-0 PMC6883579

[61] Lu J, Rincon N, Wood DE, Breitwieser FP, Pockrandt C, Langmead B, et al. Metagenome analysis using the Kraken software suite. Nat Protoc. 2022;17(12):2815–39. 36171387 10.1038/s41596-022-00738-y PMC9725748

[62] Lu J, Breitwieser FP, Thielen P, Salzberg SL. Bracken: Estimating species abundance in metagenomics data. PeerJ Comput Sci. 2017;3:e104 10.7717/peerj-cs.104 PMC12016282 40271438

[63] Beule L, Karlovsky P. Improved normalization of species count data in ecology by scaling with ranked subsampling (SRS): application to microbial communities. PeerJ. 2020;8:e9593. 32832266 10.7717/peerj.9593 PMC7409812

[64] Wilson N, Zhao N, Zhan X, Koh H, Fu W, Chen J, et al. MiRKAT: kernel machine regression-based global association tests for the microbiome. Bioinformatics (Oxford, England). 2021;37(11):1595–7. 33225342 10.1093/bioinformatics/btaa951 PMC8495888

[65] Segata N, Izard J, Waldron L, Gevers D, Miropolsky L, Garrett WS, et al. Metagenomic biomarker discovery and explanation. Genome Biol. 2011;12(6):R60. 21702898 10.1186/gb-2011-12-6-r60 PMC3218848

[66] Lin H, Peddada SD. Analysis of compositions of microbiomes with bias correction. Nat Commun. 2020;11(1):3514. 32665548 10.1038/s41467-020-17041-7 PMC7360769

[67] Lin H, Peddada SD. Multigroup analysis of compositions of microbiomes with covariate adjustments and repeated measures. Nat Methods. 2024;21(1):83–91. 38158428 10.1038/s41592-023-02092-7 PMC10776411

[68] Law CW, Chen Y, Shi W, Smyth GK. Voom: precision weights unlock linear model analysis tools for RNA-seq read counts. Genome Biol. 2014;15(2):R29. 24485249 10.1186/gb-2014-15-2-r29 PMC4053721

[69] Oshlack A, Emslie D, Corcoran LM, Smyth GK. Normalization of boutique two-color microarrays with a high proportion of differentially expressed probes. Genome Biol. 2007;8(1):R2. 17204140 10.1186/gb-2007-8-1-r2 PMC1839120

[70] Phipson B, Lee S, Majewski IJ, Alexander WS, Smyth GK. Robust hyperparameter estimation protects against hypervariable genes and improves power to detect differential expression. Ann Appl Stat. 2016;10(2):946–63. 28367255 10.1214/16-AOAS920 PMC5373812

[71] Ritchie ME, Phipson B, Wu D, Hu Y, Law CW, Shi W, et al. Limma powers differential expression analyses for RNA-sequencing and microarray studies. Nucleic Acids Res. 2015;43(7):e47. 25605792 10.1093/nar/gkv007 PMC4402510

[72] Fernandes AD, Reid JNS, Macklaim JM, McMurrough TA, Edgell DR, Gloor GB. Unifying the analysis of high-throughput sequencing datasets: characterizing RNA-seq, 16S rRNA gene sequencing and selective growth experiments by compositional data analysis. Microbiome. 2014;2(1):15. 24910773 10.1186/2049-2618-2-15 PMC4030730

[73] Deek RA, Ma S, Lewis J, Li H. Statistical and computational methods for integrating microbiome, host genomics, and metabolomics data. eLife. 2024;13:e88956. 38832759 10.7554/eLife.88956 PMC11149933

[74] Nelson ME, Jester DJ, Petkus AJ, Andel R. Cognitive reserve, Alzheimer’s neuropathology, and risk of dementia: a systematic review and meta-analysis. Neuropsychol Rev. 2021;31(2):233–50. 33415533 10.1007/s11065-021-09478-4 PMC7790730

[75] Gasmi Benahmed A, Gasmi A, Doşa A, Chirumbolo S, Mujawdiya PK, Aaseth J, et al. Association between the gut and oral microbiome with obesity. Anaerobe. 2021;70:102248. 32805390 10.1016/j.anaerobe.2020.102248

[76] Meng S, He X, Fu X, Zhang X, Tong M, Li W, et al. The prevalence of sarcopenia and risk factors in the older adult in China: a systematic review and meta-analysis. Front Public Health. 2024;12:1415398. 39161853 10.3389/fpubh.2024.1415398 PMC11331796

[77] Beeri MS, Leugrans SE, Delbono O, Bennett DA, Buchman AS. Sarcopenia is associated with incident Alzheimer’s dementia, mild cognitive impairment, and cognitive decline. J Am Geriatr Soc. 2021;69(7):1826–35. 33954985 10.1111/jgs.17206 PMC8286176

[78] Wu Y, Li D, Vermund SH. Advantages and Limitations of the Body Mass Index (BMI) to Assess Adult Obesity. Int J Environ Res Public Health. 2024;21(6):757. 38929003 10.3390/ijerph21060757 PMC11204233

[79] Van Eldik LJ, Carrillo MC, Cole PE, Feuerbach D, Greenberg BD, Hendrix JA, et al. The roles of inflammation and immune mechanisms in Alzheimer’s disease. Alzheimer’s & Dementia: Translational Research & Clinical Interventions. 2016;2(2):99–109. 10.1016/j.trci.2016.05.001 PMC5644267 29067297

[80] Alqahtani T, Deore SL, Kide AA, Shende BA, Sharma R, Dadarao Chakole R, et al. Mitochondrial dysfunction and oxidative stress in Alzheimer’s disease, and Parkinson’s disease, Huntington’s disease and Amyotrophic Lateral Sclerosis -an updated review. Mitochondrion. 2023;71:83–92. 37269968 10.1016/j.mito.2023.05.007

[81] Nearing JT, Comeau AM, Langille MGI. Identifying biases and their potential solutions in human microbiome studies. Microbiome. 2021;9(1):113. 34006335 10.1186/s40168-021-01059-0 PMC8132403

[82] Brooks JP, Edwards DJ, Harwich MDJ, Rivera MC, Fettweis JM, Serrano MG, et al. The truth about metagenomics: quantifying and counteracting bias in 16S rRNA studies. BMC Microbiol. 2015;15:66. 25880246 10.1186/s12866-015-0351-6 PMC4433096

[83] Nearing JT, Douglas GM, Hayes MG, MacDonald J, Desai DK, Allward N, et al. Microbiome differential abundance methods produce different results across 38 datasets. Nat Commun. 2022;13(1):342. 35039521 10.1038/s41467-022-28034-z PMC8763921

[84] Yeo K, Connell J, Bouras G, Smith E, Murphy W, Hodge JC, et al. A comparison between full-length 16S rRNA Oxford nanopore sequencing and Illumina V3–V4 16S rRNA sequencing in head and neck cancer tissues. Arch Microbiol. 2024;206(6):248. 38713383 10.1007/s00203-024-03985-7 PMC11076400

[85] Könönen E, Fteita D, Gursoy UK, Gursoy M. *Prevotella* species as oral residents and infectious agents with potential impact on systemic conditions. J Oral Microbiol. 2022;14(1):2079814. 36393976 10.1080/20002297.2022.2079814 PMC9662046

[86] Downes J, Sutcliffe IC, Booth V, Wade WG. *Prevotella maculosa* sp. nov., isolated from the human oral cavity. Int J Syst Evol Microbiol. 2007;57(Pt 12):2936–9. 18048753 10.1099/ijs.0.65281-0

[87] B Abraham S, Al-Marzooq F, Samaranayake L, Hamoudi RA, Himratul-Aznita WH, Aly Ahmed HM. Molecular analyses indicate profuse bacterial diversity in primary and post- treatment endodontic infections within a cohort from the United Arab Emirates-a preliminary study. PLoS ONE. 2024;19(7):e0305537. 39008450 10.1371/journal.pone.0305537 PMC11249272

[88] Romani Vestman N, Chen T, Lif Holgerson P, Öhman C, Johansson I. Oral microbiota shift after 12-week supplementation with *Lactobacillus reuteri* DSM 17938 and PTA 5289; a randomized control trial. PLoS ONE. 2015;10(5):e0125812. 25946126 10.1371/journal.pone.0125812 PMC4422650

[89] Diaz PI, Hoare A, Hong BY. Subgingival microbiome shifts and community dynamics in periodontal diseases. J Calif Dent Assoc. 2016;44(7):421–35. 27514154

[90] Bansal K, Chaudhary R, Mathur VP, Tewari N. Comparison of oral micro-flora in caries active and caries free Indian children using culture techniques and PCR analysis. Indian J Dent Res. 2020;31(3):420–5. 32769277 10.4103/ijdr.IJDR_39_19

[91] Chen X, Daliri EBM, Chelliah R, Oh DH. Isolation and identification of potentially pathogenic microorganisms associated with dental caries in human teeth biofilms. Microorganisms. 2020;8(10):1596. 33081291 10.3390/microorganisms8101596 PMC7603000

[92] Ordinola-Zapata R, Costalonga M, Dietz M, Lima BP, Staley C. The root canal microbiome diversity and function. A whole-metagenome shotgun analysis. Int Endod J. 2024;57(7):872–84. 36861850 10.1111/iej.13911

[93] Pilarczyk-Zurek M, Sitkiewicz I, Koziel J. The clinical view on *Streptococcus anginosus* group – opportunistic pathogens coming out of hiding. Front Microbiol. 2022;13:956677. 35898914 10.3389/fmicb.2022.956677 PMC9309248

[94] Rams TE, Feik D, Mortensen JE, Degener JE, van Winkelhoff AJ. Antibiotic susceptibility of periodontal *Streptococcus constellatus* and *Streptococcus intermedius* clinical isolates. J Periodontol. 2014;85(12):1792–8. 25102269 10.1902/jop.2014.130291

[95] Bao K, Bostanci N, Thurnheer T, Belibasakis GN. Proteomic shifts in multi-species oral biofilms caused by *Anaeroglobus geminatus*. Sci Rep. 2017;7(1):4409. 28667274 10.1038/s41598-017-04594-9 PMC5493653

[96] Chugal N, Wang JK, Wang R, He X, Kang M, Li J, et al. Molecular characterization of the microbial flora residing at the apical portion of infected root canals of human teeth. J Endod. 2011;37(10):1359–64. 21924182 10.1016/j.joen.2011.06.020 PMC3415298

[97] Tyrrell KL, Citron DM, Warren YA, Nachnani S, Goldstein EJC. Anaerobic bacteria cultured from the tongue dorsum of subjects with oral malodor. Anaerobe. 2003;9(5):243–6. 16887710 10.1016/S1075-9964(03)00109-4

[98] Murad CF, Sassone LM, Faveri M, Hirata RJ, Figueiredo L, Feres M. Microbial diversity in persistent root canal infections investigated by checkerboard DNA-DNA hybridization. J Endod. 2014;40(7):899–906. 24935532 10.1016/j.joen.2014.02.010

[99] Qudeimat MA, Alyahya A, Karched M, Behbehani J, Salako NO. Dental plaque microbiota profiles of children with caries-free and caries-active dentition. J Dent. 2021;104:103539. 33248211 10.1016/j.jdent.2020.103539

[100] Nibali L, Sousa V, Davrandi M, Spratt D, Alyahya Q, Dopico J, et al. Differences in the periodontal microbiome of successfully treated and persistent aggressive periodontitis. J Clin Periodontol. 2020;47(8):980–90. 32557763 10.1111/jcpe.13330

[101] Kolenbrander PE, Andersen RN, Moore LV. Coaggregation of *Fusobacterium nucleatum*, *Selenomonas flueggei*, *Selenomonas infelix*, *Selenomonas noxia*, and *Selenomonas sputigena* with strains from 11 genera of oral bacteria. Infect Immun. 1989;57(10):3194–203. 2777378 10.1128/iai.57.10.3194-3203.1989 PMC260789

[102] Marchesan J, Jiao Y, Schaff RA, Hao J, Morelli T, Kinney JS, et al. TLR4, NOD1 and NOD2 mediate immune recognition of putative newly identified periodontal pathogens. Mol Oral Microbiol. 2016;31(3):243–58. 26177212 10.1111/omi.12116 PMC4713362

[103] Chávez De Paz LE, Molander A, Dahlén G. Gram-positive rods prevailing in teeth with apical periodontitis undergoing root canal treatment. Int Endod J. 2004;37(9):579–87. 15317560 10.1111/j.1365-2591.2004.00845.x

[104] Dioguardi M, Alovisi M, Crincoli V, Aiuto R, Malagnino G, Quarta C, et al. Prevalence of the genus *propionibacterium* in primary and persistent endodontic lesions: a systematic review. J Clin Med. 2020;9(3):739. 32182900 10.3390/jcm9030739 PMC7141369

[105] Signoretti FGC, Endo MS, Gomes BPFA, Montagner F, Tosello FB, Jacinto RC. Persistent extraradicular infection in root-filled asymptomatic human tooth: scanning electron microscopic analysis and microbial investigation after apical microsurgery. J Endod. 2011;37(12):1696–700. 22099908 10.1016/j.joen.2011.09.018

[106] Robertson D, Smith AJ. The microbiology of the acute dental abscess. J Med Microbiol. 2009;58(2):155–62. 19141730 10.1099/jmm.0.003517-0

[107] Zhang C, Yang Z, Hou B. Diverse bacterial profile in extraradicular biofilms and periradicular lesions associated with persistent apical periodontitis. Int Endod J. 2021;54(9):1425–33. 33711170 10.1111/iej.13512

[108] Mayanagi G, Sato T, Shimauchi H, Takahashi N. Detection frequency of periodontitis-associated bacteria by polymerase chain reaction in subgingival and supragingival plaque of periodontitis and healthy subjects. Oral Microbiol Immunol. 2004;19(6):379–85. 15491463 10.1111/j.1399-302x.2004.00172.x

[109] Marchesan JT, Morelli T, Moss K, Barros SP, Ward M, Jenkins W, et al. Association of Synergistetes and cyclodipeptides with periodontitis. J Dent Res. 2015;94(10):1425–31. 26198391 10.1177/0022034515594779

[110] Colombo APV, Boches SK, Cotton SL, Goodson JM, Kent R, Haffajee AD, et al. Comparisons of subgingival microbial profiles of refractory periodontitis, severe periodontitis, and periodontal health using the human oral microbe identification microarray. J Periodontol. 2009;80(9):1421–32. 19722792 10.1902/jop.2009.090185 PMC3627366

[111] Chen T, Marsh PD, Al-Hebshi NN. SMDI: an index for measuring subgingival microbial dysbiosis. J Dent Res. 2022;101(3):331–8. 34428955 10.1177/00220345211035775 PMC8982011

[112] Vieira Colombo AP, Magalhães CB, Hartenbach FARR, Martins do Souto R, Maciel da Silva-Boghossian C. Periodontal-disease-associated biofilm: a reservoir for pathogens of medical importance. Microbial pathogenesis. 2016;94:27–34. 26416306 10.1016/j.micpath.2015.09.009

[113] Siqueira JFJ, Silva WO, Romeiro K, Gominho LF, Alves FRF, Rôças IN. Apical root canal microbiome associated with primary and posttreatment apical periodontitis: a systematic review. Int Endod J. 2024;57(8):1043–58. 38634795 10.1111/iej.14071

[114] Wang Y, Wang S, Wu C, Chen X, Duan Z, Xu Q, et al. Oral microbiome alterations associated with early childhood caries highlight the importance of carbohydrate metabolic activities. mSystems. 2019;4(6):e00450-19. 31690590 10.1128/mSystems.00450-19 PMC6832018

[115] Usuga-Vacca M, Marquez-Ortiz RA, Castellanos JE, Martignon S. Association of root biofilm bacteriome with root caries lesion severity and activity. Caries Res. 2024;58(1):39–48. 38128496 10.1159/000535923

[116] Pinto KP, Barbosa AFA, Silva EJNL, Santos APP, Sassone LM. What is the microbial profile in persistent endodontic infections? A scoping review. J Endod. 2023;49(7):786-798.e7. 37211309 10.1016/j.joen.2023.05.010

[117] Sisk-Hackworth L, Ortiz-Velez A, Reed MB, Kelley ST. Compositional data analysis of periodontal disease microbial communities. Front Microbiol. 2021;12:617949. 34079525 10.3389/fmicb.2021.617949 PMC8165185

[118] Giacomini JJ, Torres-Morales J, Dewhirst FE, Borisy GG, Mark Welch JL. Site specialization of human oral *Veillonella* species. Microbiol Spectr. 2023;11(1):e0404222. 36695592 10.1128/spectrum.04042-22 PMC9927086

[119] Papapanou PN, Park H, Cheng B, Kokaras A, Paster B, Burkett S, et al. Subgingival microbiome and clinical periodontal status in an elderly cohort: the WHICAP ancillary study of oral health. J Periodontol. 2020;91 Suppl 1(Suppl 1):S56-67. 32533776 10.1002/JPER.20-0194 PMC8324315

[120] Chen X, Hu X, Fang J, Sun X, Zhu F, Sun Y, et al. Association of oral microbiota profile with sugar-sweetened beverages consumption in school-aged children. Int J Food Sci Nutr. 2022;73(1):82–92. 34000955 10.1080/09637486.2021.1913102

[121] Fan X, Monson KR, Peters BA, Whittington JM, Um CY, Oberstein PE, et al. Altered salivary microbiota associated with high-sugar beverage consumption. Sci Rep. 2024;14(1):13386. 38862651 10.1038/s41598-024-64324-w PMC11167035

[122] Baty JJ, Stoner SN, Scoffield JA. Oral commensal streptococci: gatekeepers of the oral cavity. J Bacteriol. 2022;204(11):e0025722. 36286512 10.1128/jb.00257-22 PMC9664950

[123] Nobbs A, Kreth J. Genetics of sanguinis-group streptococci in health and disease. Microbiol Spectr. 2019;7(1):10.1128/microbiolspec.gpp3-0052-2018. 30681069 10.1128/microbiolspec.gpp3-0052-2018 PMC11590441

[124] Huang X, Browngardt CM, Jiang M, Ahn SJ, Burne RA, Nascimento MM. Diversity in antagonistic interactions between commensal oral streptococci and *Streptococcus mutans*. Caries Res. 2018;52(1–2):88–101. 29258070 10.1159/000479091 PMC5828942

[125] Caufield PW, Schön CN, Saraithong P, Li Y, Argimón S. Oral lactobacilli and dental caries: a model for niche adaptation in humans. J Dent Res. 2015;94(9 Suppl):110S-S118. 25758458 10.1177/0022034515576052 PMC4547204

[126] Dumitriu S, Bancescu G, Murea A, Skaug N. Isolation and speciation of *Prevotella* strains from periodontal abscesses. Roumanian Arch Microbiol Immunol. 1998;57(1):5–10. 9745330

[127] Albaghdadi SZ, Altaher JB, Drobiova H, Bhardwaj RG, Karched M. In vitro characterization of biofilm formation in *Prevotella* species. Frontiers in Oral Health. 2021;2:724194. 35048047 10.3389/froh.2021.724194 PMC8757683

[128] Arias-Moliz MT, Pérez-Carrasco V, Uroz-Torres D, Santana Ramos JD, García-Salcedo JA, Soriano M. Identification of keystone taxa in root canals and periapical lesions of post-treatment endodontic infections: next generation microbiome research. Int Endod J. 2024;57(7):933–42. 38357799 10.1111/iej.14046

[129] Veras EL, Castro Dos Santos N, Souza JGS, Figueiredo LC, Retamal-Valdes B, Barão VAR, et al. Newly identified pathogens in periodontitis: evidence from an association and an elimination study. J Oral Microbiol. 2023;15(1):2213111. 37261036 10.1080/20002297.2023.2213111 PMC10228317

[130] Henne K, Rheinberg A, Melzer-Krick B, Conrads G. Aciduric microbial taxa including Scardovia wiggsiae and Bifidobacterium spp. in caries and caries free subjects. Anaerobe. 2015;35(Pt A):60–5. 25933689 10.1016/j.anaerobe.2015.04.011

[131] Selvakumar DR, Krishnamoorthy S, Venkatesan K, Ramanathan A, Abbott PV, Angambakkam Rajasekaran P. Active bacteria in carious dentin of mandibular molars with different pulp conditions: an in vivo study. J Endod. 2021;47(12):1883–9. 34534554 10.1016/j.joen.2021.08.018

[132] Park DH, Park OJ, Yoo YJ, Perinpanayagam H, Cho EB, Kim K, et al. Microbiota association and profiling of gingival sulci and root canals of teeth with primary or secondary/persistent endodontic infections. J Endod. 2024;50(8):1124–33. 38768706 10.1016/j.joen.2024.04.016

[133] Toh H, Hayashi JI, Oshima K, Nakano A, Takayama Y, Takanashi K, et al. Complete Genome Sequence of Bifidobacterium dentium Strain JCM 1195T, Isolated from Human Dental Caries. Genome Announc. 2015;3(2):e00284-e315. 25858847 10.1128/genomeA.00284-15 PMC4392159

[134] Wen ZT, Huang X, Ellepola K, Liao S, Li Y. Lactobacilli and human dental caries: more than mechanical retention. Microbiology. 2022;168(6):00119. 10.1099/mic.0.001196 PMC10233465 35671222

[135] Visentin D, Gobin I, Maglica Ž. Periodontal pathogens and their links to neuroinflammation and neurodegeneration. Microorganisms. 2023;11(7):1832. 37513004 10.3390/microorganisms11071832 PMC10385044

[136] Aja E, Mangar M, Fletcher HM, Mishra A. *Filifactor alocis*: recent insights and advances. J Dent Res. 2021;100(8):790–7. 33719654 10.1177/00220345211000656 PMC8261852

[137] Gambin DJ, Vitali FC, Casanova KAS, DE Carli JP, Mazzon RR, Gomes BPFdeA, et al. Prevalence of species of yellow, purple and green microbial complexes in endo-perio lesions: a systematic review. Braz Oral Res. 2024;38:e048. 38922208 10.1590/1807-3107bor-2024.vol38.0048 PMC11376618

[138] Issilbayeva A, Kaiyrlykyzy A, Vinogradova E, Jarmukhanov Z, Kozhakhmetov S, Kassenova A, et al. Oral microbiome stamp in Alzheimer’s disease. Pathogens. 2024;13(3):195. 38535538 10.3390/pathogens13030195 PMC10975384

[139] Panzarella V, Mauceri R, Baschi R, Maniscalco L, Campisi G, Monastero R. Oral health status in subjects with amnestic mild cognitive impairment and Alzheimer’s disease: data from the Zabút aging project. J Alzheimers Dis. 2022;87(1):173–83. 32508326 10.3233/JAD-200385 PMC9277678

[140] Taati Moghadam M, Amirmozafari N, Mojtahedi A, Bakhshayesh B, Shariati A, Masjedian Jazi F. Association of perturbation of oral bacterial with incident of Alzheimer’s disease: a pilot study. J Clin Lab Anal. 2022;36(7):e24483. 35689551 10.1002/jcla.24483 PMC9279996

[141] Antezack A, Etchecopar-Etchart D, La Scola B, Monnet-Corti V. New putative periodontopathogens and periodontal health-associated species: a systematic review and meta-analysis. J Periodontal Res. 2023;58(5):893–906. 37572051 10.1111/jre.13173

[142] Qiu C, Zhou W, Shen H, Wang J, Tang R, Wang T, et al. Profiles of subgingival microbiomes and gingival crevicular metabolic signatures in patients with amnestic mild cognitive impairment and Alzheimer’s disease. Alzheimers Res Ther. 2024;16(1):41. 38373985 10.1186/s13195-024-01402-1 PMC10875772

[143] Zheng J, Wittouck S, Salvetti E, Franz CMAP, Harris HMB, Mattarelli P, et al. A taxonomic note on the genus *Lactobacillus*: description of 23 novel genera, emended description of the genus *Lactobacillus* beijerinck 1901, and union of Lactobacillaceae and Leuconostocaceae. Int J Syst Evol Microbiol. 2020;70(4):2782–858. 32293557 10.1099/ijsem.0.004107

[144] Del Pilar Angarita-Díaz M, Fong C, Medina D. Bacteria of healthy periodontal tissues as candidates of probiotics: a systematic review. Eur J Med Res. 2024;29(1):328. 38877601 10.1186/s40001-024-01908-2 PMC11177362

[145] Ahmed HS. The impact of prevotella on neurobiology in aging: deciphering dendritic cell activity and inflammatory dynamics. Mol Neurobiol. 2024;61(11):9240–51. 38613648 10.1007/s12035-024-04156-x

[146] Bonnechère B, Amin N, van Duijn C. What are the key gut microbiota involved in neurological diseases? A systematic review. Int J Mol Sci. 2022;23(22):13665. 36430144 10.3390/ijms232213665 PMC9696257

[147] Rousée JM, Bermond D, Piémont Y, Tournoud C, Heller Y, Kehrli P, et al. Dialister pneumosintes associated with human brain abscesses. J Clin Microbiol. 2002;40(10):3871–3. 12354905 10.1128/JCM.40.10.3871-3873.2002 PMC130909

[148] Issa E, Salloum T, Tokajian S. From normal flora to brain abscesses: a review of *Streptococcus intermedius*. Front Microbiol. 2020;11:826. 32457718 10.3389/fmicb.2020.00826 PMC7221147

[149] Deng S, Zhu H, Li Y, Zhao F, Ocak U, Gong Y. An unusual case report of brain abscess caused by *prevotella loescheii* identified using the metagenomic next-generation sequencing. IDCases. 2020;20:e00758. 32337158 10.1016/j.idcr.2020.e00758 PMC7176937

[150] Sandoe JAT, Struthers JK, Brazier JS. Subdural empyema caused by Prevotella loescheii with reduced susceptibility to metronidazole. J Antimicrob Chemother. 2001;47(3):366–7. 11222578 10.1093/jac/47.3.366

[151] Santonocito S, Polizzi A, Isola G. The Impact of Diet and Nutrition on the Oral Microbiome. Adv Exp Med Biol. 2025;1472:53–69. 40111685 10.1007/978-3-031-79146-8_4

[152] Veseli A, Alidema D, Veseli K, Breznica E, Veseli E, Behluli D, et al. The impact of systemic drugs on the oral and gut microbiome: a narrative review. Georgian Med News. 2025;363:179–83. 40884384

[153] Hisamatsu D, Masuoka H, Takeshige-Amano H, Kurokawa R, Ogata Y, Suda W, et al. Acetylcholinesterase inhibitors considerably affect the salivary microbiome in patients with Alzheimer’s disease. iScience. 2025;28(6):112593. 40487442 10.1016/j.isci.2025.112593 PMC12144413

[154] DGN e. V. & DGPPN e. V. (Eds.) S3 Guideline Dementia, Version 5.2, 17.07.2025, retrieved from: https://register.awmf.org/de/leitlinien/detail/038-013. Accessed on 28.10.2025.

[155] van Zwieten A, Tennant PWG, Kelly-Irving M, Blyth FM, Teixeira-Pinto A, Khalatbari-Soltani S. Avoiding overadjustment bias in social epidemiology through appropriate covariate selection: a primer. J Clin Epidemiol. 2022;149:127–36. 35662623 10.1016/j.jclinepi.2022.05.021

[156] Iniesta M, Vasconcelos V, Sanz M, Herrera D. Supra- and subgingival microbiome in gingivitis and impact of biofilm control: a comprehensive review. Antibiotics (Basel). 2024;13(6):571. 38927237 10.3390/antibiotics13060571 PMC11200379

[157] Holmer J, Aho V, Eriksdotter M, Paulin L, Pietiäinen M, Auvinen P, et al. Subgingival microbiota in a population with and without cognitive dysfunction. J Oral Microbiol. 2021;13(1):1854552. 33537116 10.1080/20002297.2020.1854552 PMC7833025

[158] Hedberg ME, Moore ERB, Svensson-Stadler L, Hörstedt P, Baranov V, Hernell O, et al. Lachnoanaerobaculum gen. nov., a new genus in the Lachnospiraceae: characterization of Lachnoanaerobaculum umeaense gen. nov., sp. nov., isolated from the human small intestine, and Lachnoanaerobaculum orale sp. nov., isolated from saliva, and reclassifi. Int J Syst Evol Microbiol. 2012;62(Pt 11):2685–90. 22228654 10.1099/ijs.0.033613-0 PMC3541798

